# Nanoemulsion: An Emerging Novel Technology for Improving the Bioavailability of Drugs

**DOI:** 10.1155/2023/6640103

**Published:** 2023-10-28

**Authors:** Preeti, Sharda Sambhakar, Rohit Malik, Saurabh Bhatia, Ahmed Al Harrasi, Chanchal Rani, Renu Saharan, Suresh Kumar, Geeta, Renu Sehrawat

**Affiliations:** ^1^Banasthali Vidyapith, Vanasthali Road, Aliyabad, Rajasthan 304022, India; ^2^Gurugram Global College of Pharmacy, Haily Mandi Rd, Farukh Nagar, Haryana 122506, India; ^3^School of Health Sciences, University of Petroleum and Energy Studies, Dehradun, Uttarakhand, India; ^4^Natural & Medical Sciences Research Centre, University of Nizwa, Birkat Al Mauz, Oman; ^5^Maharishi Markandeswar Deemed to be University, Mullana, Ambala, Haryana 133203, India; ^6^Ganpati Institute of Pharmacy, Yamunanagar, Haryana 135102, India; ^7^School of Medical & Allied Sciences, K R Mangalam University, Gurugram, Haryana 122103, India

## Abstract

The pharmaceutical sector has made considerable strides recently, emphasizing improving drug delivery methods to increase the bioavailability of various drugs. When used as a medication delivery method, nanoemulsions have multiple benefits. Their small droplet size, which is generally between 20 and 200 nanometers, creates a significant interfacial area for drug dissolution, improving the solubility and bioavailability of drugs that are weakly water-soluble. Additionally, nanoemulsions are a flexible platform for drug administration across various therapeutic areas since they can encapsulate hydrophilic and hydrophobic medicines. Nanoemulsion can be formulated in multiple dosage forms, for example, gels, creams, foams, aerosols, and sprays by using low-cost standard operative processes and also be taken orally, topically, topically, intravenously, intrapulmonary, intranasally, and intraocularly. The article explores nanoemulsion formulation and production methods, emphasizing the role of surfactants and cosurfactants in creating stable formulations. In order to customize nanoemulsions to particular medication delivery requirements, the choice of components and production techniques is crucial in assuring the stability and efficacy of the finished product. Nanoemulsions are a cutting-edge technology with a lot of potential for improving medication bioavailability in a variety of therapeutic contexts. They are a useful tool in the creation of innovative pharmaceutical formulations due to their capacity to enhance drug solubility, stability, and delivery. Nanoemulsions are positioned to play a crucial role in boosting medication delivery and enhancing patient outcomes as this field of study continues to advance.

## 1. Introduction

Nanoemulsions, also known as nanometric-sized emulsions, are fine water-in-oil (w/o) and oil-in-water (o/w) dispersions of two immiscible fluids, as opposed to the milky-white hue concomitant with coarse dispersion. These 20–200 nm droplets are stabilized by adding the appropriate amphiphilic emulsifiers or emulsifiers. Consequently, nanoemulsions are also known as mini-emulsions. Due to kinetic stability, nanoemulsions (NE) are stable on heterogeneous systems, in contrast to microemulsions (ME). Although nanoemulsions are unique due to their extended physical constancy and are also known as “potential thermodynamic stability,” they do not appear to aggregate or flocculate. The history of nanoemulsions can be traced back to the early 20th century when researchers first began experimenting with colloidal systems. Initial work focused on macroemulsions and microemulsions, but it laid the groundwork for the development of nanoemulsions. Nanoemulsions as a distinct category of emulsions gained significant attention in the 1990s. Researchers started to explore their unique properties, such as their extremely small droplet sizes, typically ranging from 20 to 200 nanometers. This period marked a shift toward understanding the potential applications of nanoemulsions, particularly in the pharmaceutical and food industries [1–5].

**Figure 1:**
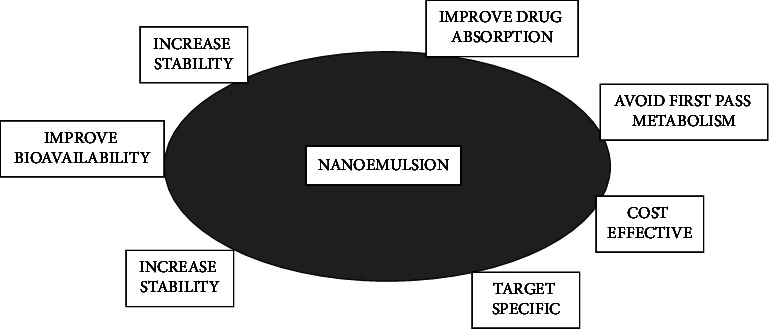
Advantages of nanoemulsions.

The following benefits explain why nanoemulsion is appealing in both the personal care and cosmetics industry and in healthcare (Figure 1) [6–25].

1. Nanoemulsion can be produced with lower concentrations of emulsifier (3–10%) than ME, which needs a high concentration (20%).
2. Nanoemulsion helps in the effective transportation of active substances through a semipermeable membrane, and due to the large surface area, penetration increases in the emulsion system.
3. Besides preventing droplet flocculation, nanoemulsions' small globule size additionally avoids larger droplet flocculation. This enables the system to survive in solitude without being divided.
4. Tiny droplets or globules in a nanoemulsion are responsible for the reduction in gravitational forces and Brownian motion. Consequently, there is no creaming or sedimentation while the product is being stored.
5. Nanoemulsions are simple to make and do not require a lot of energy to create. Nanoemulsion formulations are said to improve the reproducibility of the plasma concentration profile and bioavailability.
6. Nanoemulsions are super solvents because they include both hydrophilic and lipophilic drugs.
7. When the active ingredient is enclosed within a nanoemulsion formulation, the medicine is protected against environmental variables including pH hydrolysis and oxidation.
8. Nanoemulsions can be formed as gels, creams, foams, aerosols, and sprays, among other dosage forms. Additionally, they may be given orally, topically, intravenously, intrapulmonary, intranasally, and intramuscularly. In comparison to micelles dispersion, nanoemulsions have a higher solubilization capacity, and they are more thermo-kinetically stable.
9. It helps avoid hepatic first-pass metabolism because it is an oil/lipid-based drug delivery mechanism.
10. Nanoemulsion can also effectively mask the metallic and bitter tastes of medications that might induce unpleasant side effects such as nausea and vomiting.
11. Nanoemulsions can be useful as an alternative to liposomes and vesicles (which have poor stability), and they can occasionally be built to form lamellar liquid crystalline encircling globules.

Despite these tools, there is still a somewhat unimportant perception of the creation, production, construction, and handling of nanoemulsions. This impression is primarily attributable to the reality that traditional notions of the formation of emulsion and stability barely get extended. The proof for current perception is built up by this collective insufficiency. This study focuses on the nanoemulsion concept as a novel delivery system for poorly aqueous soluble drug candidates to enhance their bioavailability through encapsulation into oil/lipid for the management of numerous diseases, such as hypertension, diabetes, and cancer disease, and reduces the dose-related side effect of the drugs.

A wide range of advantages and standout benefits are provided by creating nanoemulsions for diverse drug delivery methods, greatly enhancing the efficacy and adaptability of pharmaceutical formulations. Transdermal gel formulations are examples of nonnanoemulsion formulations compared to nanoemulsion-based formulations that show a significant improvement in drug bioavailability. Nanoemulsions, distinguished by their nanoscale droplets, offer increased solubility for medications with low water solubility and a large interfacial area for drug dissolution. Higher drug-loading capacities as a result of this benefit enable the delivery of a larger variety of therapeutic agents. Nanoemulsions can increase medication penetration through the skin in the case of transdermal gels, resulting in a quicker beginning of action and better therapeutic results [26]. Additionally, nanoemulsions have outstanding stability, which shields drugs from deterioration and extends the shelf life of pharmaceuticals. This characteristic is particularly important when discussing transdermal gels since consistent medication administration depends on the formulation's ability to hold up over time.

The differences between transdermal gels with and without nanoemulsions highlight the advantages of the latter. Gels made from nanoemulsions have a larger drug-loading capacity, better skin penetration, and a lower risk of skin irritation. They make it possible to precisely regulate the kinetics of medication release, which improves patient compliance and therapeutic efficiency. Additionally, nanoemulsions can be administered orally, intravenously, or topically with great flexibility. Due to their adaptability, they are useful in a variety of medication delivery applications and can meet various patient demands.

## 2. Types of Nanoemulsion

Depending on the relative composition and dispersal of the more ubiquitous continuous phase and the internally distributed phases, nanoemulsions were categorized into biphasic (O/W or W/O) or multiple nanoemulsions. A nanoemulsion droplet quantity and overall durability are determined by the phase volume ratio (Φ), which also reflects the relative relevance of the internal and exterior phases that make up the nanoemulsion. The phase that is present in a greater volume typically evolves the exterior phase. The interaction of the many components that make up the nanoemulsion must be approximated to ascertain the kind of nanoemulsion that is generated under the specified parameters. O/W emulsification is favored when the emulsifier is hydrophilic or vice versa, i.e., if the emulsifier is lipophilic. Typically, an emulsifier's polar area functions as a better coalescence barrier than its hydrocarbon region.

## 3. Theories of Nanoemulsion Formulations

To investigate the mechanism of nanoemulsion production and stability, numerous methods have been employed. Some ideas (mixed film theories) emphasize the development of the interface film and the creation of extremely poor interfacial stress, whereas others (solubilization theories) emphasize the monophasic environment of the many nanoemulsions. Here are a few of these theories [27, 28].

### 3.1. Mixed Film Theories

It explains the concept of a duplex picture (i.e., displaying diverse features on both the oil and water sides) and the twisting of the interface to create o/w or w/o microemulsions. The emulsifier and coemulsifiers as oil-in-water contact create a complicated layer. This led to an extremely low level of oil-in-water interfacial tension. It was planned for the mixed interfacial layer to be fluid and dual in an environment with two-dimensional dispersion pressure, *πi*, which defined the interfacial tension, *γi*, by the following equation:

(1) $$\begin{array}{c} \gamma i=\frac{\gamma o}{w-\pi i}, \end{array}$$

where *γ* o/w symbolizes the oil-water interfacial tension [27].

### 3.2. Solubilization Theories

The idea of normal and inverse micelles is explained by the theory. The team led by Shinoda and Friberg debated on nanoemulsions as being thermodynamically established monophasic solutions of spherical micelles that are either w/o swollen or both. Oil-water emulsifier and coemulsifier quaternary phase diagrams are created [27, 28].

### 3.3. Thermodynamic Treatments

The degree to which the emulsifier reduces the surface tension between oil-water interfaces can be viewed as a determinant of the free energy of the nanoemulsion formulation. Entropy has changed in such a way that

(2) $$\begin{array}{c} ∆\text{Gf}=\gamma ∆G-TS, \end{array}$$

where ∆Gf is the free energy of development, *γ* represents interfacial tension at the oil-water interface, ∆*A* is the change in the interfacial area caused by nanoemulsification, *T* represents the temperature, and ∆*S* represents change in the system's entropy. [27, 28]

## 4. Formulation Consideration for Nanoemulsion

To develop nanoemulsion, a variety of semisynthetic oily esters, triglycerides, partial glycerides, and nonionic ester emulsifiers are commonly used. The main factor to take into account when choosing suitable excipients for lipid formulation is their ability to solubilize the entire dose in a volume adequate for unit oral administration. The type of oil-emulsifier mixture, the concentration, the ratio of the emulsifier, and the conditions or temperature at which excipients are chosen play important roles in emulsification. These facts are further corroborated by the discovery that only very specific combinations of pharmacological excipients may produce efficient emulsifying systems.

The excipients should be chosen from the USFDA's list of “GRAS” (generally recognized as safe) excipients or from other inactive ingredients that have been authorized and published by regulatory bodies. Drug release properties must not change over the course of the formulation's shelf life, and the drug must be both physically and chemically stable in the formulation. The main excipients used in emulsifying systems are lipids/oils emulsifiers and coemulsifiers [29, 30]. A few examples of diverse excipients in use are shown in Table 1.

## 5. Components of Nanoemulsion

### 5.1. Oil/Lipid

In o/w emulsions, the formulation of nanoemulsions typically contains 5–20% oil/lipid globules, while it can occasionally be much bigger (about 70%). To create nanoemulsions, re-esterified fractions from various sources including coconut oil [32], sesame oil [33], rice bran oil [34], safflower oil [35], soybean oil [36–39], and cottonseed oil [40], often categorized as short chain, medium chain, or long chain triglycerides, are used either individually or in combination. Vitamin E (D-tocopherol) has been commonly used as a lipid carrier in the development of nanoemulsions [21, 38, 39]. Nanoemulsions for topical, parenteral, and oral administration have also been made with oleic acid and ethyl oleate. The oils are chosen on the basis of how well they can dissolve drug molecules. When nanoemulsions are used for oral delivery, the oil phase of the nanoemulsions' ability to solubilize drugs is more important. You can use the oil either alone or in combination. Although the latter is preferred and safe, medium and long-chain triglyceride oils have been employed as oil phases with varying degrees of saturation. To emulsify the medication, a combination of oils and triglycerides may be employed. A number of synthetic lipids, such as Caproyl 90, triacetin, isopropyl myristate, oleic acid, palm oil esters, corn oil, olive oil, isopropyl palmitate, LabrafilMM44CS, Maisine 35-1, Miglyol 812, Captex 200, Captex 355, and Captex 8000, are often used in the production of nanoemulsions [11–13].

### 5.2. Emulsifiers

Emulsifiers are amphiphilic chemicals that reduce the interfacial tension in nanoemulsions, stabilize them, and inhibit droplet aggregation. At the oil-water interface, they frequently quickly adsorb and generate steric, electrostatic, or dual electro-steric stability. Lecithin (phosphatidylcholine), which is often obtained from egg yolk or soybean, is commonly used as an emulsifier in nanoemulsions [40]. In commercial parenteral preparations, emulsifiers such as polyoxyl 35 castor oil (Cremophor EL) and sodium deoxycholate (bile salt) have been employed. Other often used products include Solutol HS-15 (polyoxyethylene-660-hydroxy stearate), polyoxyethylene sorbitan monolaurate 20, 40, 60, and 80 (Tweens), sorbitan monolaurate 20, 40, 60, and 80 (Spans), and others. Nonionic emulsifiers are often advised due to their lower hazardous potential and low critical micelle concentration when compared to other ionic emulsifiers, and nonionic emulsifiers are thought to improve the *in vivo* stability of an oral or parenteral o/w nanoemulsion. Critical packing and hydrophile-lipophile balance (HLB) must also be taken into consideration while choosing an emulsifier. Emulsifiers with high HLB values (8–18) are used to produce o/w nanoemulsions, while emulsifiers with low HLB values (3–6) are used to produce w/o nanoemulsions. After being diluted with water, a stable nanoemulsion is produced by using the proper ratio of low and high HLB emulsifiers. Emulsifiers utilized in nanoemulsions formulation must be harmless and compatible with the final product in terms of taste, odor, and chemical stability. To ensure maximum stability, they must also develop a suitable zeta potential and viscosity in the system [3]. The common emulsifiers for different types of emulsions are listed in Table 1.

### 5.3. Coemulsifier/Cosolvent

Coemulsifiers must be introduced in very small amounts to create nanoemulsions. The majority of coemulsifier is made of (C3–C8) short- and medium-chain alcohols. They help to reduce interfacial tension and increase the interface's fluidity in the nanoemulsion. They enhance hydrocarbon tail mobility, which enhances the oil's diffusion in this region. Alcohols enhance these two contrast phases' miscibility as they are divided between oil and aqueous phases. Butanol, ethanol, propylene glycol, and isopropyl alcohol are some of the coemulsifiers that are most frequently utilized. Numerous coemulsifiers are employed as coemulsifiers in formulations are shown in Table 1 because of their acceptability to increase permeability.

## 6. Construction of Pseudoternary Phase Diagram

**Figure 2:**
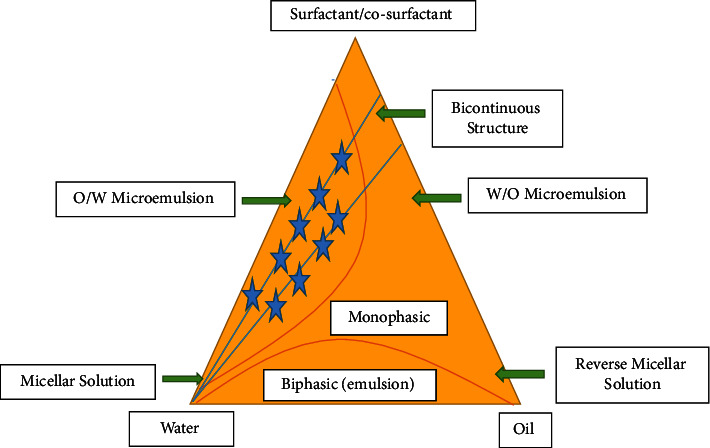
Constructed pseudoternary phase diagram showing microemulsion/nanoemulsion regions.

The initial concentration of the constituents is determined using the water titration method at room temperature by building pseudoternary phase diagrams in the nanoemulsion system [40]. Different ratios of the weight of the emulsifier and coemulsifier are used to produce various phase diagrams. These ratios are selected with increasing concentrations of coemulsifier relative to emulsifier and emulsifier relative to coemulsifier in order to thoroughly examine the phase diagrams. For each phase diagram for a specific weight ratio of emulsifier to coemulsifier, the ratios of oil to the mixture of emulsifier and coemulsifier are altered. Drop-by-drop water is added to the oil, emulsifier, and coemulsifier mixtures as they are moderately magnetically agitated. Visual observations are done for nanoemulsions that are transparent and flow readily. An artificial ternary phase diagram shows that the first axis represents the aqueous phase, the second represents the oil phase, and the third represents S_mix_ (emulsifier: coemulsifier) at a predetermined weightiness ratio as shown in Figure 2 [41, 42].

## 7. Factors Affecting the Selection of Excipients for Nanoemulsions

**Figure 3:**
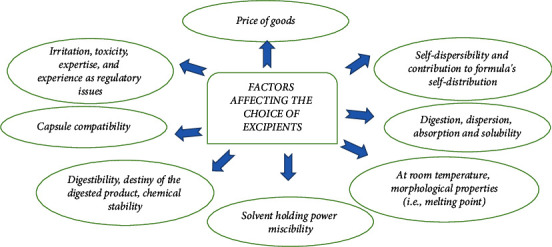
Main factor affecting the choice of excipients for nanoemulsion.

There are various factors through which choices of excipients get affected which are shown in Figure 3 [35, 43].

## 8. Methods of Preparation of Nanoemulsion Formulations

**Figure 4:**
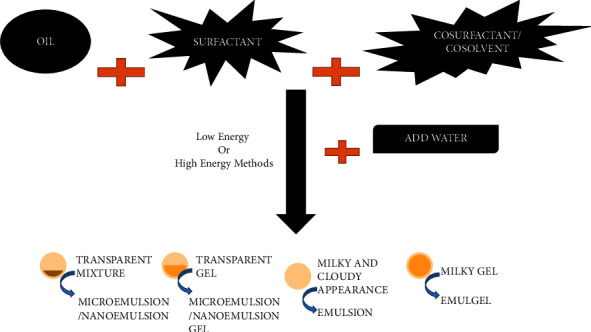
Emulsion formulation consideration and approaches as microemulsion/nanoemulsion, microgel/nanogel, emulsion, and emulgel formation.

NEs can be prepared by two methods: (1) low-energy method and (2) high-energy method (Figure 4).

### 8.1. Low-Energy Methods

Low-energy emulsification techniques use less power to produce nanoemulsion particles and are more energy-efficient since they utilize the systems' intrinsic chemical energy and only require gentle stirring as shown in Figure 3. The hydrophilic-lipophilic balance of the used oil, the emulsifier-coemulsifier mixture, operational temperature, and the accumulative behavior of drug, oil, emulsifier, coemulsifier/cosolvent, and aqueous phase, were all taken into consideration when developing these methods. Phase inversion emulsification and self-emulsification, also known as spontaneous emulsification, are two low-energy emulsification techniques. These methods' primary traits are minimal energy consumption and the production of extremely small globules or droplets [5, 44–47].

#### 8.1.1. Spontaneous Emulsification

There are three steps to it: a homogenous organic solution encompassing oil, a lipophilic emulsifier, a water-soluble cosolvent, and hydrophilic emulsifiers are first prepared as the process's initial step. Additionally, a continuous magnetic stirring process is used to produce an o/w nanoemulsion, and the aqueous phase is removed with a reduced evaporation pressure process [40].

#### 8.1.2. Phase Inversion Temperature Method (Self-Nanoemulfication Method)

It involves the natural bending of the emulsifier, which uses a heating process during emulsification that transforms the dispersed phase into the dispersion phase and *vice versa*. Changes in temperature and composition are two factors that affect spontaneous curvature. In this, phase transitions occur along the emulsification path, resulting in the production of fine dispersions through the application of chemical energy. Variations in temperature at constant composition can cause phase transitions. The effectiveness of this approach depends on how nonionic emulsifiers change solubility as a function of temperature [40].

#### 8.1.3. Phase Inverse Composition Method (Self-Nanoemusilfication Method)

By gradually adding water to an oil-emulsifier solution while gently stirring and maintaining a steady temperature, it is possible to produce kinetically stable nanoemulsions having droplets size 50 nm. Nanoemulsion produced by the spontaneous nanoemulsification process has high kinetic energy and durable colloidal stability but is not stable thermodynamically [40]. Phase inversion emulsification systems can be divided into two classes: transitional phase inversion (TPI) and catastrophic phase inversion (CPI) methods. TPI methods involve phase inversion temperature (PIT) and phase inversion composition (PIC), while CPI methods use the emulsion inversion point (EIP) [3–5, 9].

Catastrophic Phase Inversion (CPI): When the dispersed phase is constantly added, it aggregates with other drops of dispersed phase to produce bicontinuous/lamellar structural phases. A rapid alteration in a system's behavior based on altering circumstances is referred to as a catastrophe. The coalescence rate is high when the emulsifier is large in the dispersed phase, resulting in rapid phase inversion, which is required for catastrophic phase inversion to take place [5].

Emulsion Inversion Point (EIP) Method: Phase inversion occurs in the EIP technique via CPI processes. The CPI is brought on by altering the fragmented quantity of the dispersed phase. When the water phase is added to the oil-emulsifier mixture, the system starts behaving as a w/o nanoemulsion, the extra quantity of water is added while stirring constantly, water droplets interact with one another, and the phase reversal point is reached, resulting in the creation of bicontinuous or lamellar structures. An intermediary bicontinuous microemulsion is used to amplify the phase inversion process from a w/o to an o/w system. The droplet size produced in a nanoemulsion is determined by the process variables, such as the amount of water added, the stirring rate, and the amount of oil supplied [4].

### 8.2. High-Energy Method

In order to provide strong disruptive forces for size reduction during high-energy emulsification, mechanical equipment is required. Microfluidizers, homogenizers, and ultrasonicators can provide these forces, but they are expensive and produce high working temperatures, which are inappropriate for drugs that are thermolabile.

#### 8.2.1. High-Pressure Homogenization Methods

The process produces NEs from a high-pressure homogenizer/piston homogenizer with very fine particle sizes (up to 1 nm). A high-pressure homogenizer forces two liquids (oily phase and aqueous phase) through a tiny inlet hole at an incredibly high pressure to create dispersion [40].

#### 8.2.2. Microfludization

Microfludization is a unique mixing technique that simultaneously reduces particle size by attrition, impact, hydraulic shear, impingement, severe cavitation, and turbulence. This utilizes a microfluidizer device. Using a high-pressure positive displacement pump (500 to 20000 psi), the formulation is driven into the interaction chamber, which is made up of minuscule, repeated “microchannels,” producing dispersity and incredibly thin particles in the submicron range. To manufacture homogenous NEs, the procedure is done numerous times to get the required particle size [40].

#### 8.2.3. Piston Pump Homogenizer

A high-pressure homogenizer/piston homogenizer used in the process generates NEs with extremely small particle sizes (up to 1 nm). To achieve dispersion, an extremely high-pressure homogenizer pushes two liquids (oily phase and aqueous phase) via a minute inlet hole [48].

#### 8.2.4. Ultrasonication Method

An emulsion of microscale droplets that have been premixed is agitated by ultrasonic waves to produce NEs. This technique uses sonotrodes known as sonicator probes to deliver energy. It contains piezoelectric quartz crystal, which responds to an alternating electric voltage by contracting and expanding. Cavitation takes place as the sonicator's tip makes contact with the liquid, causing mechanical vibration. The collapse of vapor holes in a liquid is known as cavitation. Since emulsion may be made directly using ultrasound, it is typically employed in laboratories to make emulsion in droplets as thin as 0.2 micrometers. [40]. Ultrasonication employs the least amount of energy as compared to other high-energy procedures.

## 9. Formulation Characterization

It is necessary to evaluate and characterize these multicomponent lipidic formulations via *in vitro*, *ex vivo*, and *in vivo* measures. To characterize and assess the viability of the nanoemulsion formulation process, a variety of techniques have been used. Due to the limitations of each technique, it is challenging to characterize a formulation in its whole, but complete knowledge of the formulation is necessary for its effective commercial exploitation. Conductivity, viscosity, and dielectric constant provide valuable data at the macroscopic level. The following characteristics commonly define formulations.

### 9.1. Visual Appearance

A calibrated glass cylinder or transparent glass cylinder can be used to analyze the homogeneity and color of the appearance at equilibrium [43].

### 9.2. Color, Odor, and Taste

These characteristics are specifically essential for formulations that are meant to be taken orally. Variations in taste, particularly of active components, are usually caused by alterations in globule size, crystal habit, and subsequent globule size distribution. The taste, odor, and color of particular ingredient can vary which suggest the chemical incompatibility [43].

### 9.3. Density

The specific gravity or density of nanoemulsion formulation is two crucial parameters. A decrease in the formulation's density is typically a sign that there is trapped air inside its composition. Density at certain temperatures can be determined with high-precision hydrometers. [43].

### 9.4. pH

The pH value of a particular formulation is tested with a pH meter at specific temperatures once sedimentation equilibrium has been reached in order to reduce “pH drift,” or the buildup of suspended particles on the electrode surface. It is not recommended to add neutral electrolytes to the formulation's outer phase in order to regulate the pH because they affect the suspension's physical stability [43].

### 9.5. Osmolarity Measurements

Nanoemulsion osmolarity is determined using a micro-osmometer using the freezing point method. This is done by transferring 100 *μ*l of nanoemulsion into a microtube and taking measurements [49]. When Morsi et al. used a pH meter to measure the acetazolamide nanoemulsion's pH, they found that it ranged from 4.9 to 5.5, indicating that it was suitable and nonirritating for use in the eye [50].

### 9.6. Phase Behavior Studies

Characterization and ingredient optimization are the goals of this investigation (emulsifier and oil and aqueous phases). In order to ascertain the phases of nanoemulsions and dispersibility, a study is typically required in cases of micro/NEs formulation prepared by the PIT method and the self-nanoemulsification methods.

### 9.7. Emulsion Droplet Polarity

It is a crucial element in determining the effectiveness of emulsification. The polarization of the oil globules is significantly influenced by the HLB, chain length, degree of unsaturation of the fatty acid, hydrophobic part molecular weight, and content of the emulsifier. The medicinal compound's attraction for oil or water, as well as the nature of forced formation, is represented by polarity [25].

### 9.8. Thermodynamic Stability Studies

Three steps are taken to complete these studies.

First, a heating-cooling cycle with varied temperature conditions is used to see how this affects the stability of the nanoemulsion. By keeping the formulation for at least 24 hours at each temperature, nanoemulsions are subjected to six cycles between 4°C and 40°C. For the following experiment, centrifugation, those preparations that are constant at all these conditions will be chosen.

The second centrifugation involves spinning the prepared nanoemulsion at 5000 rpm for 30 minutes while observing breaking, creaming, and phase separation. After centrifugation, those nanoemulsions that are stable and have not displayed any signs of instability are subsequently put through a freeze-thaw cycle.

Third, the study exposes nanoemulsion formulations to three cycles of the freeze-thaw cycle at various temperatures between −21° and +25°C. Formulations that pass the test and do not exhibit any signs of instability are thought to have strong stability [49, 50].

Dispersibility tests are carried out with the formulation that passed the aforementioned thermodynamic test once these tests are finished to determine the effectiveness of self-emulsification [49, 51].

### 9.9. Dispersibility Study

This study, which was conducted using a typical USP XXII dissolution apparatus, was done to assess the effectiveness of self-emulsification of nanoemulsion formulations. 500 ml of the dissolution medium is filled with 2.1 ml of each formulation, and the temperature is kept at 37±0.5°C. For gentle agitation, a stainless-steel paddle is circulated at 50 rpm. A basic dissolution paddle made of stainless steel revolves at 50 rpm to provide light agitation. Using the grading method presented below [50], the *in vitro* performance is evaluated visually of the nanoemulsion formulations.

  Grade A: Nanoemulsions seem clear or bluish and form quickly within one minute
  Grade B: Nanoemulsions formed quickly but are to some extent less clear; they have a bluish-white appearance
  Grade C: Fine milky emulsions form with in less than 20 minutes
  Grade D: Emulsions have a cloudy appearance in color, appear slightly greasy, and take longer to form (>2 min)
  Grade E: Large oil globules may be visible on the nanoemulsion's surface, which exhibits either weak or insignificant emulsification

### 9.10. Identification Test for Nanoemulsion

#### 9.10.1. Conductivity Measurement

By measuring conductivity with conductometers, we can determine the emulsion type and whether the microphase is continuous with oil or with water. This technique can also identify phase inverse phenomena. Water in the inner phase of the w/o type of nanoemulsion prevents it from conducting, whereas water in the external phase of an o/w type of nanoemulsion makes it highly conductible. Electrical conductance measurements are extremely helpful for identifying the dispersion phase's characteristics and for spotting phase inversion phenomena. The use of the dielectric constant in determining structural and dynamic characteristics is very important. A conductometer with two electrodes immersed in the nanoemulsion and connected to an electric source is used to measure the conductivity of the nanoemulsion. If the lamp glows during the test, the nanoemulsion is of the o/w type as water conducts the passage of current between connecting electrodes; however, if the lamp does not glow, the nanoemulsion is of w/o type because oil is in the exterior phase and oil does not conduct the current [40, 52].

#### 9.10.2. Fluorescence Test

Numerous oils show fluorescence when they are radiated with UV light. When an o/w type nanoemulsion under a microscope is exposed to UV light, the fluorescence appears as spots instead of an entire field while in the case of w/o type, i.e., vice versa [40].

#### 9.10.3. Dye Solubilization

A water-soluble dye disperses in o/w globules while remaining soluble disperses in the aqueous phase of w/o globules. Similarly, an oil-soluble dye is dispersed in the w/o globules but is soluble in the o/w globule's oily phase [53]. A water-soluble dye will spread evenly when added to an o/w nanoemulsion, but when used with a w/o emulsion, the dye will only persist in the dispersed phase. A microscopic study of the emulsion will reveal this [40]. By including a water-soluble dye called eosin yellow into the formulation and observing it under a microscope, Laxmi et al. performed a dye solubilization test on an artemether containing nanoemulsion. They found that the oily dispersed phase was left unlabeled although the continuous phase was dye-labeled, confirming that the produced nanoemulsion was an o/w type.

#### 9.10.4. Dilutability

The purpose of the dilutability test is to demonstrate that a dispersion phase can be introduced to a nanoemulsion in greater amounts without negatively impacting its stability. Since w/o nanoemulsions cannot be diluted with water, they undergo an inversion phase and become o/w nanoemulsions. In contrast, O/W nanoemulsions may not undergo the same inversion phase. Only oil can be used to dilute a w/o nanoemulsion [40, 53]. When Laxmi et al. tested the dilutability of a nanoemulsion by adding water to it, they found no evidence of phase inversion or precipitation, concluding that their formulation was stable [54].

#### 9.10.5. Percent Transmittance

The percent transmittance of a prepared formulation is calculated using a UV spectrophotometer at a specified wavelength and distilled water as a blank. A nanoemulsion is deemed to be transparent if it's percent transmittance is proven to be greater than 99% [55]. A nanoemulsion of amphotericin B with a percent transmittance of >97% was reported by Harika and Debnath [56].

#### 9.10.6. Interfacial Tension

Measuring the interfacial tension allows researchers to better understand how nanoemulsions form and behave. Particularly, the presence of emulsifying agents between the aqueous and oil phases in equilibrium corresponds to phase behavior at incredibly low interfacial tension values. A spinning-drop apparatus is used to evaluate very low interfacial tension. Spinning a drop of low-density phase inside a cylinder of high-density phase helps to determine its shape, allowing for the measurement of interfacial tensions [53].

#### 9.10.7. Determination of Encapsulation Efficacy

A weighed amount of the formulation is ultrasonically determined, i.e., dispersed in an organic solvent to ascertain how much drug is contained within it, and the drug is then extracted into an appropriate buffer. By spectrophotometrically analyzing the extract at *λ*_max_ the maximum appropriate dilutions against an appropriate blank, the drug content is calculated. These equations can be used to calculate the entrapment efficiency (EE) and loading efficiency (LE) of the drug. Drug LE is defined as drug content in the obtained product (mg)/total product weight (mg)×100 [61], whereas drug EE is defined as drug content in the obtained product (mg)/total drug added (mg) ×100. Drug content could also be assessed using reverse-phase high-performance liquid chromatography (HPLC) [58].

#### 9.10.8. Viscosity and Refractive Index Measurement

A rotational viscometer of the Brookfield type can be used to measure the viscosity of LBFs with different compositions at different temperatures and shear rates. The samples to be tested must be submerged in it prior to testing, and a thermo bath must be used to keep the sample temperature at 37°C. To ensure reproducibility at a specific temperature, the viscometer used must be properly calibrated to measure the apparent viscosity of the suspension at equilibrium. Abbe's refractometer can be used to calculate the refractive index. It provides information on how isotropic the formulation is [43, 59].

#### 9.10.9. Particle Size and Polydispersity Index (PDI) Determination

By monitoring variations in scattering light caused by the Brownian movement of particles over time, Malvern Zetasizer is used to determine nanoemulsion particle size and PDI. According to the PCS hypothesis, small particles travel more quickly than large particles, according to the PCS hypothesis. The solution's submicron particles warp the laser beam. Particle diffusion, which is influenced by particle size, causes significant variations in the intensity of laser light scattering around the constant angle mean value. The size of the particle can be determined using a line width distribution histogram generated by an estimated photoelectron time-correlation function. Double-distilled water is combined with a weighed amount of the formulation to make a homogenous dispersion that must be used right away to gauge the particle size and PDI. The PDI of a monodisperse system is zero, but the PDI of a polydisperse particle dispersion is one [59].

#### 9.10.10. Morphology Characterization of Nanoemulsion

As illustrated in flowchart 1, the morphology can be characterized using either an electron microscope or a light scattering approach. In a dynamic light-scattering spectrophotometer, the dynamic light scattering at 90 degrees is measured using a neon laser with a wavelength of 632 nm. The gadget has a built-in computer that processes the data. The more recent technique for particle detection is known as Photon Correlation Spectroscopy (PCS). Some of the commonly used microscopy techniques for morphology characterization are discussed in detail here:

1. Scanning electron microscopy (SEM): Nanoemulsion size, size distribution, and morphology can be determined using SEM data (self-emulsifying powder). However, drying and sorting samples could cause the specimen to shrink and change in appearance [60, 61]. Additionally, some biomolecule specimens that are nonconductive when scanned by an electron beam have a tendency to charge up and refract the beam, causing inaccuracies in the imaging. To get around this, the sample preparation technique calls for an ultrathin coating of an electrically conductive substance on the molecule [61]. In order to scan the attached groups to nanoemulsion surfaces using an electron microscope, a cryogenic freezing technique is frequently required. Furthermore, due to the restricted number of particle samples in the scanning zone, partial assessments of the size distribution of heterogeneous samples are required in SEM [62].
2. Transmission electron microscope (TEM): It is one of the utmost adequate and commonly applied techniques for describing nanoemulsions in electron microscopy. A conceptual resolution to the atomic dimension level (1 nm) and a clear image of nanoemulsions are both provided by TEM[63]. An extremely thin sample specimen is subjected to an incoming electron beam, which causes the bombarded electrons to interact with the sample specimen and get distorted into either elastically scattered electrons or unscattered electrons [64]. The distance between the specimen and the objective lens, as well as the distance between the objective lens and its planer image, is the primary determining factor of TEM magnification [64]. The high three-dimensional resolution of TEM further improves the structural and morphological characteristics of nanoemulsions, and it can be combined with a variety of analytical techniques. It is fascinating to note that wet TEM can be utilized to assess the particle size, agglomeration, dispersion, and dynamic displacement of nanoemulsions in an aqueous environment [65].
3. Atomic force microscopy (AFM): The size, form, dispersion, sorption, and aggregation of nanoemulsions are currently being examined using a new technology called AFM. Many scanning modes utilized in AFM research include stagnant mode (also known as a noncontact mode), contact mode, tapping mode (also called intermittent sample contact mode), and dynamic mode [66–68]. Because it can image biomolecules without apparent damage to diverse intrinsic surfaces, AFM is becoming more and more important. The foremost asset of AFM is its capacity to image a variability of nanoemulsions in aqueous fluids at the subnanometer scale [69].
4. Dynamic light scattering (DLS): Using radiation scattering technology, the DLS approach is also utilized to describe the physicochemical characteristics of nanoemulsions (self-emulsifying powder), such as biomolecular conformation, aggregation state, and shape [70, 71]. DLS is based on the idea that for a certain scattering angle, Rayleigh scattering—a transient fluctuation in scattered light's intensity—is created by the Brownian motion of the size of the molecule or particle, and that the smaller the size, the less light is scattered [71]. The scattered lights combined with beneficial and harmful interventions cause the intensity to fluctuate [72].

#### 9.10.11. Zeta Potential Determination (Surface Charges)

Using a Malvern Zetasizer instrument, which measures the zeta potential (ZP) of the nanoemulsion preparation, one can ascertain the surface charge that is present on the particle or globules. The ZP predicts the stability of the dispersion and how the physicochemical characteristics of the drug, emulsifier, coemulsifier, polymers, vehicle, and other electrolytes present in the formulation will affect its value. This measurement of the particle surface charge provides information about the repelling forces between particles, drops, and globules. It also provides information about how these forces are absorbed. Nanoemulsion is diluted for the purpose of evaluating zeta potential value which is determined by the oil globules electrophoretic mobility. ZP should normally increase to a value over 30 mV in order to establish stable NE by preventing the nanodroplets from coalescing and flocculating [70].

#### 9.10.12. Fourier Transform Infra-Red Spectroscopy (FTIR)

The assessment of drug-excipient interactions, cross-linking, polymerization, and drug entrapment in the formulation can all be done using FTIR analysis. Additionally, it is employed for molecular fingerprinting and the identification of functional groups together with their modes of attachment. At low temperatures, molecules are found in their ground state. Infra-red spectroscopy is based on measuring the energy differences (delta E) between the ground state and excited state of the molecules. These molecules get excited to a higher energy state by absorbing radiant radiation [52].

The most popular method for analyzing spectral bands to reveal the conjugation of nanoemulsion carriers is FTIR spectroscopy [71–73]. In the present, a developed method known as attenuated total reflection (ATR)-FTIR spectroscopy is used to analyze the structure of chemical species at an interface using the effects of total internal reflection in light via IR [74, 75]. ATR-FTIR offers IR absorption spectra for evaluating things like changes in surface characteristics as well as pinpointing the chemical characteristics of polymer surfaces, among other things [75].

#### 9.10.13. *In Vitro* Drug Release Study

This study is done to evaluate the nanoemulsion formulation's *in vivo* performance. USP dissolution apparatus at 37 ± 0.5°C and 50 rpm stirring speeds are typically used in this study. Samples are taken out at regular intervals, and an equal amount of dissolution medium is added at each sample withdrawal time. Then, samples are diluted appropriately, and their absorbance is measured using spectrophotometry at a specific wavelength. Nanoemulsion or solid nanoparticles containing the drug were dispersed in a buffer solution, introduced into the dialysis bag, and put in a buffer-containing flask. These absorbance data are used in combination with the calibration curve to determine the percentage of drug release at various time intervals [59, 76].

#### 9.10.14. *In Vitro* Skin Permeation Studies

Permeation tests are conducted both *in vitro* and *ex vivo* using the Keshary-Chien diffusion cell. The abdominal skin of mature male rats weighing 250 ± 10 g is commonly used in this study. A portion of rat skin is set in the diffusion cells' donor and receiver compartment. Fresh water receiver chambers containing 20% ethanol are continuously swirling at 300 rpm and maintained at a constant temperature of 37°. The formulas are placed in the donor room. At certain intervals, such as 2, 4, 6, and 8 hours, a predetermined volume (0.5 ml) of the receiver compartment's solution was removed for examining gas chromatography. Each time, an equivalent amount of fresh solution was added right away to replace the withdrawn volume. Three times the same sample is utilized. Cumulative adjustments are used to calculate the total quantity of drugs that entered the rat skin at each point in time, and the findings are plotted against the passage of time. In a steady state, the slope of the plot is used to calculate drug penetration rates [77]. Harwansh et al. used the Franz diffusion cell to investigate the transdermal penetration of glycyrrhizin through human cadaver skin. The standard gel formulation was found to be less permeable to the skin than the nanoemulsion formulation [78].

#### 9.10.15. *In Vivo* Studies

*In vivo* research can be carried out by employing an animal model that is appropriate for the specific and chosen activity. Srilatha et al. investigated the antidiabetic activity of glipizide nanoemulsion in a hyperglycemia model in which rats were first given an intraperitoneal injection of streptozocin solution to induce diabetes. The formulation was then administered to the diabetic model of rats, and pharmacodynamic parameters were studied. For up to 12 hours, they observed lower blood glucose levels [51]. By conducting pharmacokinetic experiments on the nanoemulsion, Chouksey et al. analyzed the *in vivo* activity of atorvastatin and found that it had a higher bioavailability than the pure drug [79]. With the appropriate methods, the full potential of nanoemulsions as a tool for efficient medicine delivery could be realized. Parameters such as quality assurance and quality control should be of the utmost importance with such a precise method of delivery system; as a result, the evaluation tests shall be carried out attentively.

#### 9.10.16. Stability Studies

This study is performed to estimate the stability of the drug candidate when it is exposed to different drug substances, i.e., when it is exposed to different environmental factors such as humidity, temperature, and light. According to the references of the ICH (International conference on harmonization), a stability study of the nanoemulsion is carried out after the preparation has been stored for at least 730 days in a freeze-dried or dispersed state. These studies are conducted under low temperature (25°F/60°RH), freeze (−20°F), and refrigeration (5°F) temperature storage conditions. The essential portion of the nanoemulsion is kept in carefully sealed glass vials, and samples are taken at predetermined intervals. Particle size, polydispersity index, entrapment efficacy, and drug release profiles, among other characteristics, were also examined [80].

#### 9.10.17. Shelf-Life Determination

The study of accelerated stability is performed to determine the nanoemulsion's shelf life while the formulation is kept at three distinctive temperatures and humidity (30°, 40°, and 50°RH) for at least 90 days. The remaining drug amount in the nanoemulsion formulation is evaluated using HPLC (high-performance liquid chromatography) investigation of samples taken at regular intervals (0, 30, 60, and 90 days) under these various settings. As controls, samples taken at the start of time are used [52]. This establishes the reaction's order, and the reaction rate constant (*K*) for deterioration is then computed from the slope lines at each high temperature using the following equation. The logarithm values of *K* are plotted against the reciprocal of absolute temperature at various raised temperatures using slope = *K*/2.303 (Arrhenius plot). *K* at 25° is calculated from this plot, and the value is then employed in the following equation to determine shelf life:

(3) $$\begin{array}{c} t_{0.9}=\frac{0.1052}{K_{25}}, \end{array}$$

where “shelf life” is well-defined as the amount of time needed for 10% of a medicine to degrade [41].

## 10. Application of Nanoemulsion: Encapsulating Drugs for Improving the Bioavailability

Recently, poor drug compounds have drawn attention. This section gives an overview of research that has increased the bioavailability of drug compounds by encapsulating them in nanoemulsions. The research on nanoemulsions created utilizing high- or low-energy techniques is the main emphasis of this review. Nanoemulsion can be produced at low cost in different pharmaceutical dosage forms, such as creams, gels, foams, sprays, and aerosols, and it can also be taken orally, topically, intravenously, intrapulmonary, intranasally, and intraocularly. Nanoemulsion is a safe and effective dosage form of subpar medication candidates for increasing bioavailability in the treatment of a variety of conditions, such as hypertension, inflammation, and cancer, and lowers the dose-related adverse impact of the drugs. There have already been several research conducted as shown in Table 2 that supports the idea that nanoemulsion is an emerging novel technology for improving the bioavailability of drugs.

## 11. Application of Nanoemulsion in Drug Delivery

### 11.1. Nanoemulsion and Drug Targeting

The fascinating application that is currently under the development involves using of nanoemulsion formulations for the delivery of controlled and targeted medication [134]. Their submicron size makes it simple to target the tumor's location. Aqueous insoluble drugs have historically been delivered by nanoemulsions, but more recently, attention has been focused on colloidal particles as a carrier for the targeted delivery of different anticancer medications, photosensitizers, neutron capture therapy agents, or diagnostic agents. A novel method of treating cancer is the production of magnetic nanoemulsions. These can spread into tissue layers of the skin with photosensitizers like Foscan®, causing hyperthermia and the subsequent production of free radicals. Photodynamic therapy, which uses this technology, can be utilized to treat cancer [135].

### 11.2. Drug Delivery via Transdermal Nanoemulsions

There has been a lot of interest in this area since it is practical to provide medications through the skin to the systemic circulation for a variety of clinical diseases [136, 137]. The parenteral method has the advantage of continuous drug-controlled distribution over a longer period of time, even when self-administration may not be possible. The patient can stop taking the medication at any time by simply removing the transdermal patch. Nanoemulsions have a pleasant feeling on the skin thanks to their transparency and fluidity. The complete absence of gastrointestinal adverse effects such as irritating gastrointestinal and bowel ulcers, which are typically linked to oral delivery, is an added benefit. For a variety of illnesses and disorders, such as cardiovascular problems, Parkinson's disease, Alzheimer's disease, anxiety, and depression, transdermal medicinal treatments have been produced. The main drawback to this form of administration is the skin's barrier, which prevents the bioactives from penetrating the body effectively. The stratum corneum, which severely limits their absorption and bioavailability, the sweat ducts, and hair follicles are the three primary routes through which drugs can enter the skin. The major objective is to improve drug targeting and pharmacokinetics. The main skin barriers must be broken down for better medication pharmacokinetics and targeting. Additionally, it is important to manage the redistribution of topically applied medications through the cutaneous blood and lymphatic system. Nano-sized emulsions can quickly enter the systemic circulation and pass through the skin's pores, channeling the substance for efficient distribution [138]. By oral administration, caffeine has been utilized to treat a variety of cancers. Caffeine nanoemulsions in water-in-oil have been created for transdermal medication delivery. These and aqueous caffeine solutions' in vitro skin permeation profiles were compared, and the nanoemulsion-loaded medicines' permeability parameters significantly increased [139].

### 11.3. Drug Delivery via Pulmonary Nanoemulsions

Very little research has been published in this field, and the nanoemulsion method has not yet been completely utilized for pulmonary drug administration [140]. As an alternative to liposomes as a gene transfer vector, emulsion systems have been developed [141]. Other researches on emulsion for gene administration (nonpulmonary route) indicated that the emulsion or DNA combination had a strong affinity than liposomal carriers [142]. Genes were administered more effectively using this stable emulsion approach compared with liposomes [143]. According to Bivas-Benita et al. [144], cationic submicron emulsions are promising DNA vaccine delivery systems to the lung because they can transfect pulmonary epithelial cells, which may result in cross-priming of antigen-presenting cells and direct activation of dendritic cells, stimulating antigen-specific T cells. As a result, the nebulization of submicron emulsions will be a new and developing research in the field of pharmaceutical sciences. However, due to the potential negative effects of oil and Emulsifiers on lung alveolar function, more research is required to formulate the inhalable submicron emulsion as a successful route of pulmonary administration (adverse interactions with lung emulsifier).

### 11.4. Delivery of Parenteral Drugs Using Nanoemulsions

This is one of the most common and efficient drug delivery methods, and it is typically used for active ingredients with low bioavailability and limited remedial indices. Because of the ability to dissolve large amounts of hydrophobics, mutual compatibility, and the potential to protect medicines from enzymatic degradation and hydrolysis, nanoemulsions are ideal carriers for parenteral administration. Furthermore, because these emulsions ensure that medications are released continuously and consistently over long periods of time, the injection dosage and their frequency can be reduced throughout the period of drug therapy. In this context, the lack of flocculation, creaming, and sedimentation, as well as the high surface area and free energy, provide clear benefits over emulsions with larger particle sizes. Furthermore, because these emulsions ensure that pharmaceuticals are released continuously and under control over long periods of time, the frequency and dosage of injections can be reduced during the course of drug therapy. The absence of flocculation, creaming, and sedimentation, combined with the high surface area and free energy, clearly outperforms emulsions with larger particle sizes in this situation. It was loaded into parenteral emulsions made using the high-energy ultrasonication method to study its pharmacokinetics and anticancer activity. This nanoemulsion treatment for colon adenocarcinoma in mice results in the suppression of higher tumors rather than a plain solution of drug treatment, indicating that drug-loaded emulsion may be a useful vehicle for medication transport in treating cancer [145]. There was no parenteral treatment available for patients due to carbamazepine's limited water solubility, a common anticonvulsant medication. Kuo et al. [77] elaborated a nanoemulsion with good *in vitro* release kinetics for intravenous administration.

### 11.5. Delivery of Ophthalmic Drugs Using Nanoemulsions

A wide range of diseases are categorized as ophthalmic ailments, including glaucoma, cataracts, dry eye syndrome, and numerous ocular infections. Due to the defensive systems of the eye, including tear film dynamics and the blood-ocular barrier, it is frequently difficult to deliver medications to the eye successfully. By encapsulating medications into nanoscale droplets, nanoemulsions are able to effectively penetrate the ocular surface and maintain a longer residence duration. Ophthalmic nanoemulsions have a number of significant uses, including the treatment of glaucoma, a major contributor to permanent blindness. Antiglaucoma medications' ocular bioavailability can be improved using nanoemulsions, enabling lower dosages and less systemic adverse effects. Furthermore, nanoemulsions are adaptable carriers for a variety of therapeutic agents utilized in ophthalmology because they may include both hydrophilic and hydrophobic medicines. Additionally, because they are more tolerable and cause less discomfort, nanoemulsion-based ophthalmic formulations can increase patient compliance. These formulations address a prevalent problem with conventional ophthalmic therapies by being frequently less viscous and easier for patients to administer [146].

### 11.6. Delivery of Intranasal Drugs Using Nanoemulsions

In addition to oral and parenteral administration routes, intranasal drug delivery systems are now recognized as an effective route for the administration of dosage forms. The nasal mucosa has been shown to be a therapeutically effective route for systemic medication administration and an effective strategy for circumventing barriers that prevent direct drug entry into the target-oriented site. This method was also painless, tolerable, and noninvasive. Due to less enzymatic activity, more immunoactive sites, and permeable epithelium layer, the nasal cavity is one of the most effective places for the delivery of drug [147]. Targeting medications for the brain poses several challenges, especially for hydrophilic and large molecular-weight medications. This is due to the impermeable properties of the endothelium, which separates the systemic circulation and acts as a blood-brain barrier [148]. The nasal mucosa's olfactory region serves as a direct link between the nose and the brain, and ailments such as Alzheimer's disease, migraine, depression, schizophrenia, Parkinson's disease, and meningitis are treated with medication-loaded nanoemulsions [149, 150]. There have been reports of risperidone nanoemulsions being developed for nasal administration [150, 151]. It is implied that this emulsion works better when taken orally rather than intravenously. Another therapeutic application for intranasal drug delivery systems is vaccine development. Immunity is produced as a result of mucosal antigen delivery, and the first intranasal vaccine is now available on the market. One of the potential delivery methods is the use of nanocarriers, which shows considerable excellence in protecting biomolecules, fostering nanocarrier interaction with mucosae, and directing antigens to lymphoid tissues. The use of nanoemulsion technology in intranasal drug delivery systems is expected to produce significant results in treating central nervous system disorders by effectively targeting medications to the brain.

## 12. Future Prospective

Since its creation, nanoemulsion has proven to be a versatile and effective new medication delivery technology. Because they have a limited capacity for solubilizing nonpolar active chemicals, nanoemulsions are being proposed for a variety of uses in pharmacies as drug delivery methods. Future applications of nanoemulsion in various therapeutic disciplines or in the creation of cosmetics for the skin or hair are quite bright. Nanoemulsions have a wide range of uses, including medication delivery, where they serve as effective carriers for bioactive and make a variety of administration methods possible. Their parenteral delivery has been used to meet nutritional needs, manage drug release, deliver vaccines, and target drugs to certain locations. There are many benefits and uses for oral medication administration using these vehicles, where the size of the droplets affects how well they are absorbed in the GIT. The application of nanoemulsions in ocular delivery, where pharmaceutical medicines are better maintained than their corresponding solutions, has also been researched. Other effective administration methods for nanoemulsified delivery systems include pulmonary and transdermal routes. Although there have not been many reports of nanoemulsion uses in other domains, these subjects have a lot of potential, including engineering, agriculture, and the chemical and physical sciences. The price of making nanoemulsions will go down as new equipment for high-pressure homogenization becomes available and manufacturers begin to compete with one another. Optimized emulsifier systems and more efficient emulsifier utilization will result from the fundamental study into the function of emulsifiers in the process of producing nanoemulsions. The ability to modify nanoemulsions for targeted distribution holds great promise in treating malignancies and in delivering drugs to the brain in the field of oncology.

## 13. Conclusion

The development and designing of nanotechnology for emulsion systems became a critical parameter for managing and/or improving therapeutic drug bioavailability. Particle size reduced to the nanometric scale exhibits some intriguing physical characteristics, such as optical transparency and abnormal elastic behavior. Nanoemulsions, useful dispersions of deformable nanoscale droplets with a range of flow characteristics and optical properties which range from opaque to nearly transparent, hold great promise in the field of nanomaterials. Furthermore, nanoemulsions are expected to play a larger role in the commercial sphere because they can often be made with a significantly low quantity of emulsifier as compared to nanostructured lyotropic microemulsion systems. The review paper highlights and gives a brief description of the recent developments in the field of nanoemulsion carrier formulations which are discovered till date. Nanoemulsions are gaining popularity as drug carrier candidate for enhancing the delivery of pharmaceutical active ingredients because they provide a number of benefits for pharmaceutical delivery. Because they are adaptable to virtually all delivery methods, they have the potential to be used in a broad range of disciplines, including cosmetics, pharmaceuticals, and biotechnology.

Nanoemulsion drug delivery systems became an advanced key tool for effective delivery of drugs and reaching a target site. Compared to most conventional dosage forms, they also provide effective bioactive material encapsulation protection and improved delivery. The potential lies in formulation specialists' inventiveness in overcoming unusual drug delivery issues including permeability and *in vivo* stability by utilizing the benefits of nanoemulsion carriers. We believe that more research is needed to fully realize the potential of nanoemulsion technology in the delivery of novel phytopharmaceuticals and tiny molecule medications. This novel method could be developed to overpower the drug candidates' limitations such as poor solubility, absorption, and miscibility with lipids found in cell membrane lining.

## Figures and Tables

**Table 1:** Various excipients widely used in nanoemulsion formulations.

Brand name/chemical definition (HLB value)	Components	Traders/manufacturers
Tween 20 (T20)/PEG-20 sorbitan monolaurate (16.7)	Emulsifier	Altas/ICI
Tween 60 (T60)/polysorbate 60 (14)	Emulsifier	Atlas/ICI
Tween 65 (T65)/PEG-20 sorbitan tristearate (11)	Emulsifier	Atlas/ICI
Tween 80 (T80)/PEG-20 sorbitan monooleate (15)	Coemulsifier	Atlas/ICI
Span 20 (S20)/sorbitan monolaurate (8.6)	Emulsifier	Atlas/ICI
Span 60 (S60)/sorbitan monostearate 4.7	Coemulsifier	Atlas/ICI
Span80 (S80)/sorbitan monooleate (4.3)	Coemulsifier	Atlas/ICI
Brij-30/PEG-4 lauryl ether (9.7)	Emulsifier	Atlas/ICI
Arlasolve DMI/Di-methyl isosorbide-	Coemulsifier	Atlas/ICI
Capmul MCM-C8/glyceryl caprylate (5-6)	Emulsifier	ABITEC
Lecithin/L-a-phosphatidylcholine (4–9)	Emulsifier	Alfa Aesar
Cerex ELS 250/PEG-25 hydrogenated castor oil (11)	Emulsifier	Auschem SpA
Akoline MCM/caprylic/capric glycerides (5-6)	Coemulsifier	Aarhuskarlshamn
Cremophor-EL, cremophor-ELP/ PEG-35 castor oil (12–14)	Emulsifier	BASF
Cremophor RH40/PEG-35 hydrogenated castor oil (13)	Emulsifier	BASF
Pluronic L44/block copolymer of ethylene oxide and propylene oxide (12–18)	Coemulsifier	BASF
Lutrol F 68/polaxomer 188 (29)	Coemulsifier	BASF
Cremophor RH 40/polyoxyl 40 hydrogenated castor oil (13)	Emulsifier	BASF
SLS/sodium lauryl sulfate (40)	Coemulsifier	Canadian Alcolac Ltd.
Carbitol/diethylene glycol monoethyl ether-	Co-emulsifier	Dow chemicals
TPGS/(Tocophersolan, D-*α*-tocopheryl PEG-1000 succinate) (13)	Emulsifier	Eastman
Labrafil M 2125 CS/PEG-6 corn oil (4)	Emulsifier	Gattefosse
Labrafil M1944CS/PEG-6 apricot kernel oil (4)	Emulsifier	Gattefosse
Labrasol/PEG-8 caprylic/capric glycerides (14)	Emulsifier	Gattefosse
Labrafac CM 10/PEG-8 caprylic/capric glycerides (>10)	Emulsifier	Gattefosse
Labrafil WL 2609 BS/PEG-8 corn oil (6-7)	Emulsifier	Gattefosse
Peceol glyceryl monooleate (3-4)	Emulsifier	Gattefosse
Plurol oleique CC 497 caprol® 6G20 hodag PGO-62/ polyglyceryl-6 dioleate (6)	Coemulsifier	Gattefosse Abitec Co Calgene
Lauroglycol 90/propylene glycol monolaurate (5)	Coemulsifier	Gattefosse
Lauroglycol FCC/propylene glycol monolaurate (4)	Coemulsifier	Gattefosse
Transcutol P/diethylene glycol mono ethyl ether-	Coemulsifier	Gattefosse
Labrafil 1944/PEG-6 apricot kernel oil (4)	Coemulsifier	Gattefosse
HCO-40/polyoxyethylene hydrogenated castor oil 40 (13)	Emulsifier	Nikkol
HCO-60/PEG-60 hydrogenated castor oil (14)	Coemulsifier	Nikkol
Emulphor El-620/ethoxylated castor oil (12–15)	Emulsifier	Rhodia

**Table 2:** A review of nanoemulsion formulation prepared for the treatment of various diseases such as cancer, hypertension, and inflammatory [81–133].

S. no	Drug class	Drug candidate	Absorbent/polymers/bioenhancers/other excipients	Lipids/oils	Emulsifier: coemulsifier	Formulation approach	Year of publication	Reference no.	Outcomes
*Anticancer drugs*									
1	Phosphoinositide 3-kinase inhibitor	Resveratrol	Hepes buffer	Neem oil	Tween 20	NE	2021	[81]	This study discovered a significant reduction in cell viability after incubating bladder T24 cancer cells with RV-loaded NEs versus free RV
2	Pyrimidine antagonists	5-Fluorouracil	Chitosan powder/curcumin	—	Span 80	NE	2022	[82]	This study shows that curcumin/5-fluorouracil was loaded in the nanocarrier and proved to be a candidate in the treatment of targeted cancer cells
3	Folate antagonist	Methotrexate	—	Olive oil	Labrasol: ethanol	NE	2020	[83]	This research shows that methotrexate-loaded nanoemulsions have a high potential for improving methotrexate-targeted lymphatic delivery
4	Microtubule damaging agents	Docetaxel	—	Olive oil	Tween 80: oleyl alcohol	NE	2018	[84]	This study shows that nebulized DNE4 has better aerosolization properties for pulmonary administration and is more particular on human lung cancer cells (A549) as compared to normal cells (MRC-5)
5	Cyclooxygenase-2 inhibitor	Etoricoxib	—	Isopropyl myristate	PEG 200 transcutol HP	NE	2021	[85]	The initiation of apoptosis/necrotic cell death and S-phase cell cycle arrests in A549 cells demonstrated the nanoemulsion's potential and cytotoxic action against lung cancer cells
6		Carvacrol	—	—	Polysorbate 80	NE	2018	[86]	Reactive oxygen species (ROS) was produced in A549 cells as a result of the carvacrol nanoemulsion, which then activated the caspase cascade and critical apoptosis regulators such as p-JNK, Bax, and Bcl2. The apoptotic potential of CANE was reversed when mitochondrial ROS was suppressed using mito-TEMPO, indicating that mitochondrial ROS is involved in cell death
7	Microtubule damaging agents	Paclitaxel	—	Oleic acid	PEG400	NE	2017	[87]	This study demonstrates that paclitaxel nanoemulsion medication exhibits larger plasma AUC0-∞ value, more cytotoxicity against MCF-7 cells, and more robust anticancer efficacy on MCF-7 tumor-bearing nude mice
8	Quinone reductase 2 (QR2) inhibitor	Quercetin	Agarose	Paraffin oil	Polyvinylpyrrolidone (PVP-K90)	NE	2021	[88]	This study demonstrates the development of a pH-responsive nanocomposite encapsulated in nanoemulsions to simultaneously enhance the loading and sustained-release of quercetin using HAp nanoparticles and nanoemulsions, resulting in improved bioavailability and potency against cancerous cells
9	Antimetastatic	Piplartine	—	Capmul PG-8	Tween 80 PEG 400	NE	2016	[89]	The piperine nanoemulsions improved the bioavailability of PL, significantly reduced the growth of melanoma tumors *in vivo*, and did not exhibit any toxicity when used for an extended period of time
10	Benzene and substituted derivatives	Benzyl isothiocyanate	—	Flaxseed oil	Tween 80, Transcutol	NE	2011	[90]	A better-performing, higher-permeability benzyl thiocyanate o/w nanoemulsion has been developed, and it inhibits cancer cell growth *in vitro*
11	Antibiotics	Doxorubicin	Cholesterol		Tween 80 PEG2000	NEG	2022	[91]	This study demonstrates that the anticancerous properties of doxorubicin's nanoemulgel technology are superior and effective against Kaposi's sarcoma, breast, and ovarian cancer
12		Astaxanthin	—	Peanut oil	D-*α*-tocopheryl polyethylene glycol succinate (TPGS)	NE	2020	[92]	This study reveals that astaxanthin nanoemulsion combined with oral chemotherapy has a favorable chemotherapeutic effect on melanoma with lung metastases under *in vivo* settings
13		Camptothecin analogue SN38	—	—	Polypropylene glycol (PPO) PEG	NE, ME	2013	[93]	This study shows that using macromolecular prodrugs (such as EZN-2208 and IMMU-135) and nanomedicine formulations, the transport of SN38 to cancer cells and tissue has been improved (such as nanoemulsions, polymeric, micelles, lipid nanocapsules and nanoparticles, and liposomes)
14		Hyaluronic acid	Phosphate buffer saline solution	—	—	NE	2021	[94]	This study shows that HA-SH polymer with phosphate buffer saline solution is used to make the nanoemulsion form of hyaluronic acid and is effective against specific cancer cell lines and shows a good oncolytic effect
15		Catechins	—	Lecithin	—	NE	2021	[95]	The DU-145 cell cycle was arrested at the S and G2/M phases by catechin nanoemulsions and extracts, which increased caspase-8, caspase-9, and caspase-3 activities
16		Zingiber	—	—	—	NE	2022	[96]	Flow cytometry was used in this study to show that ZOEO significantly increased the sub-G1 populations (cell death) in cell cycle analysis and promoted cell apoptosis in apoptotic analysis
17		Diferuloylmethane	—	Ethyl oleate	Cremophor EL 35 PEG 400	NE	2016	[97]	In comparison to free DIF, this study demonstrates that DNHLNs exhibited better bioavailability and stronger antilung cancer action
18		Citrus lemon	—	Tween 80	Tween 20 ethylene glycol	NE	2020	[98]	Citrus lemon essential oil nanoemulsion possesses an antiangiogenic tendency and selectively promotes apoptosis in A549 human lung cancer cells. In order to treat human lung cancer, CLEO-NE may be used as a secure natural anticancer compound
19	Folate antagonists	Pemetrexed	—	Castor oil	Labrasol, Tween 80 PEG 400	NE	2018	[99]	A w/o/w multiple NE was developed in this study for the simultaneous administration of PMX and QCN, and it was demonstrated that it improved oral absorption and had synergistic anticancer effects. The inhibitory effects of PMX and PMX/DCK in combination with QCN on cancer cell growth were concentration-dependent
20		Carmustine	Phosphatidylcholine	Trioleate	Cholesteryl oleate	NE	2013	[100]	The use of this innovative formulation to treat canine lymphomas was demonstrated to be safe and effective in a drug combination strategy, which motivates larger studies
21	Topoisomerase 2 inhibitor	Etoposide	—	Capryol 90	Tween 80 D-alpha-tocopherol polyethylene glycol succinate (TPGS)	NE	2020	[101]	The results of the intestinal transport mechanistic study revealed that mechanisms such as clathrin-/caveola-mediated endocytosis, macropinocytosis, and ASBT-mediated pathways contributed to the increased permeability and oral bioavailability of etoposide-loaded nanoemulsion and demonstrated an anticarcinogenic effect
22	Topoisomerase-1-inhibitor	Irinotecan	—	Ethiodized oil	Iohexol	NE	2020	[102]	The results of this study show that IRI-lipiodol nanoemulsion is effective in the treatment of metastatic colon cancer
23	Antibiotics	Bleomycins	—	Fish oil	Kolliphor	NE	2022	[103]	*In vitro* tests have shown pulmonary fibrosis to be resistant to omega-3 fatty acids with bleomycin nanoemulsion characteristics
24	Nitrogen mustards alkylating agent	Chlorambucil	—	Soyabean oil	Lecithin PEG2000	NE	2010	[104]	According to this study, lipid nanoemulsion chlorambucil could result in a noticeably better pharmacokinetic profile and therapeutic efficiency of chlorambucil compared to non-PEG-modified nanoemulsion and solution
25	Platinum coordination complexes	Cisplatin	—	Olive oil	Lecithin ethylene glycol		2021	[105]	In order to increase cisplatin's anticancer activity toward DLD1 cells, nanoemulsions work in synergy with it to improve cisplatin's bioactivity
26	Platinum coordination complexes	Oxaliplatin	—	Capryol 90	Labrasol, Transcutol HP		2020	[106]	Oral metronomic chemotherapy was performed in this study using OXA/DCK complex-loaded multiple nanoemulsion (OXA/DCK-NE), which was developed for OXA oral delivery

*Antihypertensive agents*									
26	CCB	Amlodipine	—	Oleic acid	Tween 80, Transcutol P	NE	2009	[107]	This study suggests that nanoemulsion could be used as a more effective transdermal delivery method for amlodipine
27	CCB	Amlodipine basilate	—	Labrafil M	Tween 80 ethanol	NE	2011	[108]	This study showed threefold increases in the total residence duration of NE, suggesting that NE could serve as drug carriers to boost bioavailability when compared to commercial formulations
28	CCB	Isradipine	—	Tricetin	Tween 20, Transcutol	NE	2020	[109]	This study demonstrated greater isradipine solubility and dissolution profile
29	CCB	Nitrendipine	—	Caproyl 90®	Tween 80: Transcutol P®/solutol HS-15®	Intranasal NE	2009	[110]	*In vivo* studies show improved absorption, a faster onset of action, and a relative bioavailability of 60.44%, which is higher than that of commercially available tablets and pure solution
30	CCB	Niterndipine	—	Capmul MCM: Triacetin	Kolliphor ELP, Transcutol HP	NE gel	2020	[111]	This gel enhances the inadequate penetration, and it may be employed as a viable vehicle for nitrendipine administration
31	ACE inhibitor	Captopril	Curcumin	Glyceryl monooleate	Tween 20 PEG 400	NE	2015	[112]	This study demonstrates how the NE formulation and curcumin's synergistic influence on captopril activity exacerbate the drug's poor solubility
32	ACE inhibitor	Ramipril	—	Safsol 218	Tween 80, Carbitol 18	NE	2007	[113]	This study showed that the ramipril NE formulation was optimized and may be used as a liquid unit dose form for both elderly and pediatric patients. Comparing the in vitro release to a pure suspension and a commercial formulation, it was quite substantial
33	ACE inhibitor	Ramipril	—	Safsol 218	Cremophor-EL carbitol	NE	2008	[114]	These findings suggested that ramipril is more stable in NE. Ramipril's bioavailability is 4.29 times higher in medication suspension and 1.76 times higher in tablet form
									In contrast to other formulations, the rate of degradation was slow in NE with an aqueous phase (buffered solution pH-5.0)
34	AT II receptor blockers	Candesartan cilexetil	—	Soybean oil,	Solutol HS-15: Tween 80	NE	2011	[115]	The findings of this study demonstrate that NEs are very effective formulation strategy for enhancing insoluble drug candidate oral absorption. When added to the NE, candesartan improved the AUC0-t readings by a factor of 10
35	AT II receptor blockers	Olmesartan midoxomil	—	Soyabean oil 700	Sefsol 218: Solutol HS15	NE	2014	[116]	The pharmacokinetic studies showed increased AUC of about 2.8 folds and sustained release upon oral administration
36	AT II receptor blockers	Telmisartan	Carbopol 934	Labrafil®M 2125 CS	Acrysol®EL 135 Carbitol®	NE gel	2015	[116]	The study concluded that the optimized formulation of NE gel showed higher bioavailability as compared to conventional gel and it showed greater permeation and penetration rate in *in vivo* and *in vitro*
37	AT II receptor blockers	Telmisartan	—	Oleic acid	Tween 80: PEG	NE	2017	[117]	Optimized formulation showed greater stability and drug release in comparison with conventional formulation and showed enhanced bioavailability
38	AT II receptor blockers	Telmisartan	Chitosan	Sefsol 218 and oleic acid	Tween 20: Transcutol P	Mucoadhesive nanoemulgel	2021	[118]	This study found that telmisartan mucoadhesive nanoemulgel coated with chitosan was an emerging approach for the treatment of dementia that could be delivered directly through the nose to the brain

*NSAID drugs*									
39	Salicylates	Aspirin	—	Castor oil	Lauroglycol TM90, Transcutol HP	NE	2012	[119]	Aspirin-containing new nanoemulsions and nanomultiple emulsions have anti-inflammatory and analgesic effects
40	Propionic acid derivatives	Ibuprofen	—	Olive oil	Sucrose ester laurate glycerol	NE	2020	[120]	Ibuprofen was developed in a stable nanoemulsion formulation for improved oral bioavailability
41	Propionic acid derivatives	Ketoprofen	—	Palm oil ester	Tween 80	NE	2010	[121]	A potent pharmacological component that can be used to create topical dose forms of ketoprofen is palm oil esters
42	Selective COX-2 inhibitors	Celecoxib	—	Triacetin	Cremophor-EL, Transcutol HP	NE	2010	[122]	This study demonstrates that excipients were appropriately tuned for the creation of Celecoxib's nanoemulsion formulation
43	Propionic acid derivatives	Flurbiprofen	Capryol 90	Triacetin	Tween 80 PEG400	NE, SNEDDS	2017	[123]	LCT-containing SNEDDS were thought to be the most effective at accelerating medication release
44	Fenamate	Maphenamic acid	Sodium azide	Castor oil	Kolliphor® P188 glycerol	NE	2017	[124]	Passive loading worked well for drug compounds containing mefenamic acid and was a good, simple screening technique for medication candidates with poor water solubility
45	Preferential COX-2 inhibitor	Diclofenac diethyl amine (DDEA)	Ascorbic acid, triethylamine	Oleic acid	Polysorbate 20 PEG200	NE	2020	[125]	E topical delivery of DDEA may be enhanced by incorporating the nanosystems NE and GNR into bigels
46	Preferential COX-2 inhibitor	Aceclofenac	Carbopol 940	Triacetin oil	Tween 80, Cremophor EL Transcutol HP, ethanol, PEG 400	NEG	2020	[126]	When compared to the commercial sample, the aceclofenac-nanoemulgel, which was created using Carbopol 940, showed good permeability
47	Preferential COX-2 inhibitor	Meloxicam	—	Peppermint oil	Span 80, tween 80 transcutol HP, PEG 400	SNEDDS	2021	[127]	Successfully created MX-SNELCs outperformed the typical tablet dosage form suggesting that they have the potential to be further developed into a periodontal pain management approach that is clinically acceptable
48	Acetic acid derivatives	Indomethacin-paracetamol	D-limonene		Tween 80, span 80 famotidine loaded polycaprolactone	NE	2020	[128]	This multicomponent nanoparticle may offer a platform for getting beyond the limitations of NSAIDs and combining drugs with different mechanisms of action to create effective anti-inflammatory therapy by co-encapsulating a small-sized nanoemulsion inside PCL nanoparticles
49	Enolic acid derivative	Piroxicam	—	Soyabean oil	Tween 80 transcutol HP	SNEDDS	2016	[129]	The outcomes confirm that SNEDDS is a promising method for increasing piroxicam's oral bioavailability
50	Propionic acid derivatives	Naproxen	—	Oleic acid	Sodium lauryl sulfate PEG6000	NE	2019	[130]	This study demonstrated that vehicles containing particular excipients that are known to change the characteristics of the skin were specifically shown to promote the penetration of caffeine and naproxen through the skin
51	Acetic acid derivatives	Ketorolac tromethamine	—	—	Tween 60 ethylene glycol	NE	2021	[131]	Ocular nanoemulsion preparation was developed to enhance the properties of ketorolac
52	Acetic acid derivatives	Nabumetone	—	Capmul	Gelucire	NE	2017	[132]	Solid lipid nanoparticle of nabumetone was prepared for the treatment of inflammatory diseases
53	Flavonoids	Anthocyanin	—	Carrot oil	Lecithin	NE	2019	[133]	Flavonoid anthocyanin was developed as nanoliposome and used for its anti-inflammatory action and stability is also enhanced

NE: nanoemulsion; NE gel: nanoemulgel; ME: microemulsion; SNEDDS: self-nanoemulsify drug delivery system; CCB: calcium channel blocker; ACE: angiotensin converting enzyme; AT II: angiotensin II.

## Data Availability

Information/data are collected from open source.

## Abbreviations

NE: Nanoemulsion
ME: Microemulsion
O/W: Oil-in-water
W/O: Water-in-oil
USFDA: The United States Food and Drug Administration
GRAS: Generally recognized as safe
HLB: Hydrophilic-lipophilic balance
S_mix_: Emulsifier: coemulsifier ratio
TPI: Transitional phase inversion
CPI: Catastrophic phase inversion
PIC: Phase inversion composition
EIP: Emulsion inversion point
ALTAS: Atmospheric laboratory for applications and science
ICI: Imperial Chemical Industries
EE: Entrapment efficiency
LE: Loading efficiency
LBF: Lipid-based formulation
CCB: Calcium channel blocker
PDI: Polydispersity index
PCS: Photon correlation spectroscopy
BCS: Biopharmaceutical classification system
DLS: Dynamic light scattering
SEM: Scanning electron microscopy
TEM: Transmission electron microscopy
AFM: Atomic force microscopy
ZP: Zeta potential
FTIR: Fourier transform infra-red spectroscopy
ICH: International conference on harmonization.

## References

[1] Mason T. G., Wilking J. N., Meleson K., Chang C. B., Graves S. M. (2006). Nanoemulsions: formation, structure, and physical properties. *Journal of Physics: Condensed Matter*.

[2] Ee S. L., Duan X., Liew J., Nguyen Q. D. (2008). Droplet size and stability of nano-emulsions produced by the temperature phase inversion method. *Chemical Engineering Journal*.

[3] McClements D. J. (2013). Nanoemulsion-based oral delivery systems for lipophilic bioactive components: nutraceuticals and pharmaceuticals. *Therapeutic Delivery*.

[4] Lee S. J., Choi S. J., Li Y., Decker E. A., McClements D. J. (2011). Protein-stabilized nanoemulsions and emulsions: comparison of physicochemical stability, lipid oxidation, and lipase digestibility. *Journal of Agricultural and Food Chemistry*.

[5] Bouchemal K., Briançon S., Perrier E., Fessi H. (2004). Nano-emulsion formulation using spontaneous emulsification: solvent, oil and surfactant optimisation. *International Journal of Pharmaceutics*.

[6] Yu H., Huang Q. (2012). Improving the oral bioavailability of curcumin using novel organogel-based nanoemulsions. *Journal of Agricultural and Food Chemistry*.

[7] Zhang Y., Shang Z., Gao C. (2014). Nanoemulsion for solubilization, stabilization, and in vitro release of pterostilbene for oral delivery. *AAPS PharmSciTech*.

[8] Yukuyama M. N., Ghisleni D. D. M., Pinto T. J. A., Bou‐Chacra N. A. (2016). Nanoemulsion: process selection and application in cosmetics– a review. *International Journal of Cosmetic Science*.

[9] Wang L., Li X., Zhang G., Dong J., Eastoe J. (2007). Oil-in-water nanoemulsions for pesticide formulations. *Journal of Colloid and Interface Science*.

[10] Tan S., Stanslas J., Basri M. (2015). Nanoemulsion-based parenteral drug delivery system of carbamazepine: preparation, characterization, stability evaluation and blood-brain pharmacokinetics. *Current Drug Delivery*.

[11] Sharma N., Mishra S., Sharma S., Deshpande R. D., Sharma R. K. (2013). Preparation and optimization of nanoemulsions for targeting drug delivery. *International Journal of Drug Development and Research*.

[12] Al-Edresi S., Baie S. (2009). Formulation and stability of whitening VCO-in-waternano-cream. *International Journal of Pharmaceutics*.

[13] Makidon P. E., Nigavekar S. S., Bielinska A. U. (2010). Characterization of stability and nasal delivery systems for immunization with nanoemulsion-based vaccines. *Journal of Aerosol Medicine and Pulmonary Drug Delivery*.

[14] Lala R. R., Awari N. G. (2014). Nanoemulsion-based gel formulations of COX-2 inhibitors for enhanced efficacy in inflammatory conditions. *Applied Nanoscience*.

[15] Hussain A., Samad A., Singh S. K. (2016). Nanoemulsion gel-based topical delivery of an antifungal drug: in vitro activity and in vivo evaluation. *Drug Delivery*.

[16] Nasr M., Nawaz S., Elhissi A. (2012). Amphotericin B lipid nanoemulsion aerosols for targeting peripheral respiratory airways via nebulization. *International Journal of Pharmaceutics*.

[17] Amani A., York P., Chrystyn H., Clark B. J. (2010). Evaluation of a nanoemulsion-based formulation for respiratory delivery of budesonide by nebulizers. *AAPS PharmSciTech*.

[18] Tamarkin D., Besonov A., Eini M., Danziger J. (2007). Foam prepared from nanoemulsions and uses.

[19] Mou D., Chen H., Du D. (2008). Hydrogel-thickened nanoemulsion system for topical delivery of lipophilic drugs. *International Journal of Pharmaceutics*.

[20] Khani S., Keyhanfar F., Amani A. (2016). Design and evaluation of oral nanoemulsion drug delivery system of mebudipine. *Drug Delivery*.

[21] Pawar V. K., Panchal S. B., Singh Y. (2014). Immunotherapeutic vitamin E nanoemulsion synergies the antiproliferative activity of paclitaxel in breast cancer cells via modulating Th1 and Th2 immune response. *Journal of Controlled Release*.

[22] Bhanushali R. S., Gatne M. M., Gaikwad R. V., Bajaj A. N., Morde M. A. (2009). Nanoemulsion based intranasal delivery of antimigraine drugs for nose to brain targeting. *Indian Journal of Pharmaceutical Sciences*.

[23] Ammar H. O., Salama H. A., Ghorab M., Mahmoud A. A. (2009). Nanoemulsion as a potential ophthalmic delivery system for dorzolamide hydrochloride. *AAPS PharmSciTech*.

[24] Khan A. A., Mudassir J., Mohtar N., Darwis Y. (2013). Advanced drug delivery to the lymphatic system: lipid-based nanoformulations. *International Journal of Nanomedicine*.

[25] Garti N., Frenkel M., Shwartz R. (1983). Multiple emulsions. Part II: proposed technique to overcome unpleasant taste of drugs. *Journal of Dispersion Science Andtechnology*.

[26] Singh Y., Meher J. G., Raval K. (2017). Nanoemulsion: concepts, development and applications in drug delivery. *Journal of Controlled Release*.

[27] Karasulu H. Y. (2008). Microemulsions as novel drug carriers: the formation, stability, applications and toxicity. *Expert Opinion on Drug Delivery*.

[28] Schulman J. H., Stoeckenius W., Prince L. M. (1959). Mechanism of formation and structure of micro emulsions by electron microscopy. *Journal of Physical Chemistry*.

[29] Pouton C. W., Porter C. J. H. (2008). Formulation of lipid-based delivery systems for oral administration: materials, methods and strategies. *Advanced Drug Delivery Reviews*.

[30] Singh B., Bandopadhyay S., Kapil R., Singh R., Katare O. P. (2009). Self-emulsifying drug delivery systems (SEDDS): formulation development, characterization, and applications. *Critical Reviews in Therapeutic Drug Carrier Systems*.

[31] Ghosh V., Mukherjee A., Chandrasekaran N. (2014). Eugenol-loaded antimicrobial nanoemulsion preserves fruit juice against, microbial spoilage. *Colloids and Surfaces B: Biointerfaces*.

[32] Bernardi D. S., Pereira T. A., Maciel N. R. (2011). Formation and stability of oil-in-water nanoemulsions containing rice bran oil: in vitro and in vivo assessments. *Journal of Nanobiotechnology*.

[33] Vyas T. K., Shahiwala A., Amiji M. M. (2008). Improved oral bioavailability and brain transport of Saquinavir upon administration in novel nanoemulsion formulations. *International Journal of Pharmaceutics*.

[34] Jiang S. P., He S.-N., Li Y.-L. (2013). Preparation and characteristics of lipid nanoemulsion formulations loaded with doxorubicin. *International Journal of Nanomedicine*.

[35] Anuchapreeda S., Fukumori Y., Okonogi S., Ichikawa H. (2012). Preparation of lipid nanoemulsions incorporating curcumin for cancer therapy. *Journal of nanotechnology*.

[36] Hwang Y. Y., Ramalingam K., Bienek D. R., Lee V., You T., Alvarez R. (2013). Antimicrobial activity of nanoemulsion in combination with cetylpyridinium chloride in multidrug-resistant Acinetobacter baumannii. *Antimicrobial Agents and Chemotherapy*.

[37] Abd-Elsalam K. A., Khokhlov A. R. (2015). Eugenol oil nanoemulsion: antifungal activity against Fusarium oxysporum f. sp. vasinfectum and phytotoxicity on cottonseeds. *Applied Nanoscience*.

[38] Ozturk B., Argin S., Ozilgen M., McClements D. J. (2015). Formation and stabilization of nanoemulsion-based vitamin E delivery systems using natural biopolymers: whey protein isolate and gum Arabic. *Food Chemistry*.

[39] Morais Diane J. M., Burgess J. (2014). Vitamin E nanoemulsions characterization and analysis. *International Journal of Pharmaceutics*.

[40] Jaiswal M., Dudhe R., Sharma P. K. (2015). Nanoemulsion: an advanced mode of drug delivery system. *3 Biotech*.

[41] Amselem S., Friedman D. (2019). Submicron emulsions as drug carriers for topical administration. *Submicron Emulsions in Drug Targeting and Delivery*.

[42] Shakeel F., Baboota S., Ahuja A., Ali J., Aqil M., Shafiq S. (2007). Nanoemulsions as vehicles for transdermal delivery of aceclofenac. *AAPS PharmSciTech*.

[43] Shrestha H., Bala R., Arora S. (2014). Lipid-based drug delivery systems. *Journal of pharmaceutics*.

[44] Gibaud S., Attivi D. (2012). Microemulsions for oral administration and their therapeutic applications. *Expert Opinion on Drug Delivery*.

[45] Neslihan Gursoy R., Benita S. (2004). Self-emulsifying drug delivery systems (SEDDS) for improved oral delivery of lipophilic drugs. *Biomedicine and Pharmacotherapy*.

[46] Fernandez P., André V., Rieger J., Kühnle A. (2004). Nano-emulsion formation by emulsion phase inversion. *Colloids and Surfaces A: Physicochemical and Engineering Aspects*.

[47] Ostertag F., Weiss J., McClements D. J. (2012). Low-energy formation of edible nanoemulsions: factors influencing droplet size produced by emulsion phase inversion. *Journal of Colloid and Interface Science*.

[48] Müller R., Müller R. H. (2008). Nanocrystal technology, drug delivery and clinical applications. *International Journal of Nanomedicine*.

[49] Gué E., Since M., Ropars S., Herbinet R., Le Pluart L., Malzert-Fréon A. (2016). Evaluation of the versatile character of a nanoemulsion formulation. *International Journal of Pharmaceutics*.

[50] Morsi N. M., Mohamed M. I., Refai H., El Sorogy H. (2014). Nanoemulsion as a novel ophthalmic delivery system for acetazolamide. *International Journal of Pharmacy and Pharmaceutical Sciences*.

[51] Srilatha R., Aparna C., Srinivas P., Sadanandam M. (2013). Formulation, evaluation and characterization of glipizide nanoemulsion. *Asian Journal of Pharmaceutical and Clinical Research*.

[52] Gurpreet K., Singh S. K. (2018). Review of nanoemulsion formulation and characterization techniques. *Indian Journal of Pharmaceutical Sciences*.

[53] Bhosale R. R., Osmani R. A., Ghodake P. P., Shaikh S. M., Chavan S. R. (2014). Nanoemulsion: a review on novel profusion in advanced drug delivery. *Indian Journal of Pharmaceutical and Biological Research*.

[54] Laxmi M., Bhardwaj A., Mehta S., Mehta A. (2015). Development and characterization of nanoemulsion as carrier for the enhancement of bioavailability of artemether. *Artificial Cells, Nanomedicine, and Biotechnology*.

[55] Bali V., Ali M., Ali J. (2010). Novel nanoemulsion for minimizing variations in bioavailability of ezetimibe. *Journal of Drug Targeting*.

[56] Harika K., Debnath S. (2015). Formulation and evaluation of nanoemulsion of amphotericin B. *International Journal of Novel Trends in Pharmaceutical Sciences*.

[57] Bhagav P., Upadhyay H., Chandran S. (2011). Brimonidine tartrate–eudragit long-acting nanoparticles: formulation, optimization, in vitro and in vivo evaluation. *AAPS PharmSciTech*.

[58] Singh K. K., Vingkar S. K. (2008). Formulation, antimalarial activity and biodistribution of oral lipid nanoemulsion of primaquine. *International Journal of Pharmaceutics*.

[59] Gupta S., Kesarla R., Omri A. (2013). Formulation strategies to improve the bioavailability of poorly absorbed drugs with special emphasis on self-emulsifying systems. *ISRN Pharmaceutics*.

[60] Baboota S., Shakeel F., Ahuja A., Ali J., Shafiq S. (2007). Design, development and evaluation of novel nanoemulsion formulations for transdermal potential of celecoxib. *Acta Pharmaceutica*.

[61] Hall J. B., Dobrovolskaia M. A., Patri A. K., McNeil S. E. (2007). Characterization of nanoparticles for therapeutics. *Nanomedicine*.

[62] Bootz A., Vogel V., Schubert D., Kreuter J. (2004). Comparison of scanning electron microscopy, dynamic light scattering and analytical ultracentrifugation for the sizing of poly (butyl cyanoacrylate) nanoparticles. *European Journal of Pharmaceutics and Biopharmaceutics*.

[63] Amiji M. M. (2006). *Nanotechnology for Cancer Therapy*.

[64] Williams D. B., Carter C. B. (2009). *Transmission Electron Microscopy*.

[65] Carlton C. E., Ferreira P. J. (2012). In situ TEM nanoindentation of nanoparticles. *Micron*.

[66] Hinterdorfer P., Garcia-Parajo M. F., Dufrene Y. F. (2012). Single-molecule imaging of cell surfaces using near-field nanoscopy. *Accounts of Chemical Research*.

[67] Mavrocordatos D., Pronk W., Boller M. (2004). Analysis of environmental particles by atomic force microscopy, scanning and transmission electron microscopy. *Water Science and Technology*.

[68] Picas L., Milhiet P.-E., Hernández-Borrell J. (2012). Atomic force microscopy: a versatile tool to probe the physical and chemical properties of supported membranes at the nanoscale. *Chemistry and Physics of Lipids*.

[69] Parot P., Dufrêne Y. F., Hinterdorfer P. (2007). Past, present and future of atomic force microscopy in life sciences and medicine. *Journal of Molecular Recognition*.

[70] Inagaki S., Ghirlando R., Grisshammer R. (2013). Biophysical characterization of membrane proteins in nanodiscs. *Methods*.

[71] Jiang X., Jiang J., Jin Y., Wang E., Dong S. (2005). Effect of colloidal gold size on the conformational changes of adsorbed cytochrome c: probing by circular dichroism, UV− Visible, and Infrared Spectroscopy. *Biomacromolecules*.

[72] Perevedentseva E., Cai P.-J., Chiu Y.-C., Cheng C.-L. (2011). Characterizing protein activities on the lysozyme and nanodiamond complex prepared for bio applications. *Langmuir*.

[73] Tom R. T., Samal A. K., Sreeprasad T. S., Pradeep T. (2007). Hemoprotein bioconjugates of gold and silver nanoparticles and gold nanorods: structure− function correlations. *Langmuir*.

[74] Hind A. R., Bhargava S. K., McKinnon A. (2001). At the solid/liquid interface: FTIR/ATR—the tool of choice. *Advances in Colloid and Interface Science*.

[75] Johal M. S. (2011). *Understanding Nanomaterials*.

[76] Bali V., Ali M., Ali J. (2010). Study of surfactant combinations and development of a novel nanoemulsion for minimising variations in bioavailability of ezetimibe. *Colloids and Surfaces B: Biointerfaces*.

[77] Kuo F., Subramanian B., Kotyla T., Wilson T. A., Yoganathan S., Nicolosi R. J. (2008). Nanoemulsions of an anti-oxidant synergy formulation containing gamma tocopherol have enhanced bioavailability and anti-inflammatory properties. *International Journal of Pharmaceutics*.

[78] Harwansh R. K., Patra K. C., Pareta S. K., Singh J., Rahman M. A. (2011). Nanoemulsions as vehicles for transdermal delivery of glycyrrhizin. *Brazilian Journal of Pharmaceutical Sciences*.

[79] Chouksey R., Jain A. K., Pandey H., Maithil A. (2011). In vivo assessment of atorvastatin nanoemulsion formulation. *Bulletin of Pharmaceutical Research*.

[80] Sugumar S., Mukherjee A., Natarajan C. (2015). Nanoemulsion formation and characterization by spontaneous emulsification: investigation of its antibacterial effects on Listeria monocytogenes. *Asian Journal of Pharmaceutics*.

[81] Clark M., Jepson M., Hirst B. H. (2001). Exploiting M cells for drug and vaccine delivery. *Advanced Drug Delivery Reviews*.

[82] Pourmadadi M., Ahmadi M., Abdouss M. (2022). The synthesis and characterization of double nanoemulsion for targeted Co-Delivery of 5-fluorouracil and curcumin using pH-sensitive agarose/chitosan nanocarrier. *Journal of Drug Delivery Science and Technology*.

[83] Jang J.-H., Jeong S.-H., Lee Y.-B. (2020). Enhanced lymphatic delivery of methotrexate using W/O/W nanoemulsion: in vitro characterization and pharmacokinetic study. *Pharmaceutics*.

[84] Asmawi A. A., Salim N., Ngan C. L. (2019). Excipient selection and aerodynamic characterization of nebulized lipid-based nanoemulsion loaded with docetaxel for lung cancer treatment. *Drug delivery and translational research*.

[85] Md S., Alhakamy N. A., Alharbi W. S. (2021). Development and evaluation of repurposed etoricoxib loaded nanoemulsion for improving anticancer activities against lung cancer cells. *International Journal of Molecular Sciences*.

[86] Khan I., Bahuguna A., Kumar P., Vivek K., Bajpai, Kang S. C. (2018). In vitro and in vivo antitumor potential of carvacrol nanoemulsion against human lung adenocarcinoma A549 cells via mitochondrial mediated apoptosis. *Scientific Reports*.

[87] Chen L., Chen B., Deng L. (2017). An optimized two-vial formulation lipid nanoemulsion of paclitaxel for targeted delivery to tumor. *International Journal of Pharmaceutics*.

[88] Samadi A., Pourmadadi M., Yazdian F., Rashedi H., Navaei-Nigjeh M., Eufrasio-da-silva T. (2021). Ameliorating quercetin constraints in cancer therapy with pH-responsive agarose-polyvinylpyrrolidone-hydroxyapatite nanocomposite encapsulated in double nanoemulsion. *International Journal of Biological Macromolecules*.

[89] Fofaria N. M., Qhattal H. S. S., Liu X., Srivastava S. K. (2016). Nanoemulsion formulations for anti-cancer agent piplartine—characterization, toxicological, pharmacokinetics and efficacy studies. *International Journal of Pharmaceutics*.

[90] Qhattal H. S. S., Wang S., Salihima T., Srivastava S. K., Liu X. (2011). Nanoemulsions of cancer chemopreventive agent benzyl isothiocyanate display enhanced solubility, dissolution, and permeability. *Journal of Agricultural and Food Chemistry*.

[91] D’Angelo N. A., Noronha M. A., Câmara M. C. (2022). Doxorubicin nanoformulations on therapy against cancer: an overview from the last 10 years. *Biomaterials Advances*.

[92] Haung H.-Y., Wang Y.-C., Cheng Y.-C. (2020). A novel oral astaxanthin nanoemulsion from Haematococcus pluvialis induces apoptosis in lung metastatic melanoma. *Oxidative Medicine and Cellular Longevity*.

[93] Bala V., Rao S., Boyd B. J., Prestidge C. A. (2013). Prodrug and nanomedicine approaches for the delivery of the camptothecin analogue SN38. *Journal of Controlled Release*.

[94] Deng S., Iscaro A., Zambito G. (2021). Development of a new hyaluronic acid based redox-responsive nanohydrogel for the encapsulation of oncolytic viruses for cancer immunotherapy. *Nanomaterials*.

[95] Lin Y.-H., Wang C.-C., Lin Y.-H., Chen B.-H. (2021). Preparation of catechin nanoemulsion from oolong tea leaf waste and its inhibition of prostate cancer cells DU-145 and tumors in mice. *Molecules*.

[96] Panyajai P., Chueahongthong F., Viriyaadhammaa N. (2022). Anticancer activity of Zingiber ottensii essential oil and its nanoformulations. *PLoS One*.

[97] Sun L., Wan K., Hu X. (2016). Functional nanoemulsion-hybrid lipid nanocarriers enhance the bioavailability and anti-cancer activity of lipophilic diferuloylmethane. *Nanotechnology*.

[98] Yousefian Rad E., Homayouni Tabrizi M., Ardalan P. (2020). Citrus lemon essential oil nanoemulsion (CLEO-NE), a safe cell-depended apoptosis inducer in human A549 lung cancer cells with anti-angiogenic activity. *Journal of Microencapsulation*.

[99] Pangeni R., Panthi V., Yoon I. S., Park J. (2018). Preparation, characterization, and in vivo evaluation of an oral multiple nanoemulsive system for Co-delivery of pemetrexed and quercetin. *Pharmaceutics*.

[100] Lucas S. R. R., Maranhão R. C., Guerra J. L., Coelho B. M. P., Barboza R., Pozzi D. H. B. (2015). Pilot clinical study of carmustine associated with a lipid nanoemulsion in combination with vincristine and prednisone for the treatment of canine lymphoma. *Veterinary and Comparative Oncology*.

[101] Jha S. K., Han H. S., Subedi L. (2020). Enhanced oral bioavailability of an etoposide multiple nanoemulsion incorporating a deoxycholic acid derivative–lipid complex. *Drug Delivery*.

[102] Melancon M. P., Yevich S., Avritscher R. (2021). A novel irinotecan-lipiodol nanoemulsion for intravascular administration: pharmacokinetics and biodistribution in the normal and tumor bearing rat liver. *Drug Delivery*.

[103] Galdino de Souza D., Santos D. S., Simon K. S. (2022). Fish oil nanoemulsion supplementation attenuates bleomycin-induced pulmonary fibrosis BALB/c mice. *Nanomaterials*.

[104] Ganta S., Sharma P., Paxton J. W., Baguley B. C., Garg S. (2010). Pharmacokinetics and pharmacodynamics of chlorambucil delivered in long-circulating nanoemulsion. *Journal of Drug Targeting*.

[105] Vernazza S., Dellacasa E., Tirendi S., Pastorino L., Bassi A. M. (2021). Lipoperoxide nanoemulsion as adjuvant in cisplatin cancer therapy: in vitro study on human colon adenocarcinoma DLD-1 cells. *Nanomaterials*.

[106] Choi J. U., Maharjan R., Pangeni R. (2020). Modulating tumor immunity by metronomic dosing of oxaliplatin incorporated in multiple oral nanoemulsion. *Journal of Controlled Release*.

[107] Kumar D., Aqil M., Rizwan M., Sultana Y., Ali M. (2009). Investigation of a nanoemulsion as vehicle for transdermal delivery of amlodipine. *Die Pharmazie*.

[108] Chhabra G., Chuttani K., Mishra A. K., Pathak K. (2011). Design and development of nanoemulsion drug delivery system of amlodipine besilate for improvement of oral bioavailability. *Drug Development and Industrial Pharmacy*.

[109] Ghareeb M. M. (2020). Formulation and characterization of isradipine as oral nanoemulsion. *Iraqi Journal of Pharmaceutical Sciences*.

[110] Sharma A., Singh A. P., Harikumar S. L. (2020). Development and optimization of nanoemulsion based gel for enhanced transdermal delivery of nitrendipine using box-behnken statistical design. *Drug Development and Industrial Pharmacy*.

[111] Rachmawati H., Soraya I. S., Kurniati N. F., Rahma A. (2016). In vitro study on antihypertensive and antihypercholesterolemic effects of curcumin nanoemulsion. *Scientia Pharmaceutica*.

[112] Malkawi A., Jalil A., Nazir I., Matuszczak B., Kennedy R., Bernkop-Schnürch A. (2020). Self-emulsifying drug delivery systems: hydrophobic drug polymer complexes provide a sustained release in vitro. *Molecular Pharmaceutics*.

[113] Shafiq S., Shakeel F. (2008). Enhanced stability of ramipril in nanoemulsion containing cremophor-EL: a technical note. *AAPS PharmSciTech*.

[114] Ekambaram P., Abdul Hasan Sathali A. (2011). Formulation and evaluation of solid lipid nanoparticles of ramipril. *Journal of Young Pharmacists*.

[115] Zhang Z., Gao F., Bu H., Xiao J., Li Y. (2012). Solid lipid nanoparticles loading candesartan cilexetil enhance oral bioavailability: in vitro characteristics and absorption mechanism in rats. *Nanomedicine: Nanotechnology, Biology and Medicine*.

[116] Nasr A., Gardouh A., Ghorab M. (2016). Novel solid self-nanoemulsifying drug delivery system (S-SNEDDS) for oral delivery of olmesartan medoxomil: design, formulation, pharmacokinetic and bioavailability evaluation. *Pharmaceutics*.

[117] Chin L. Y., Tan J. Y. P., Choudhury H., Pandey M., Sisinthy S. P., Gorain B. (2021). Development and optimization of chitosan coated nanoemulgel of telmisartan for intranasal delivery: a comparative study. *Journal of Drug Delivery Science and Technology*.

[118] Teaima M. H., Yasser M., Ahmed El-Nabarawi M., Ahmed Helal D. (2020). Proniosomal telmisartan tablets: formulation, in vitro evaluation and in vivo comparative pharmacokinetic study in rabbits. *Drug Design, Development and Therapy*.

[119] Tang S. Y., Sivakumar M., Ng A. M. H., Shridharan P. (2012). Anti-inflammatory and analgesic activity of novel oral aspirin-loaded nanoemulsion and nano multiple emulsion formulations generated using ultrasound cavitation. *International Journal of Pharmaceutics*.

[120] Anuar N., Sabri A. H., Bustami Effendi T. J., Abdul Hamid K. (2020). Development and characterisation of ibuprofen-loaded nanoemulsion with enhanced oral bioavailability. *Heliyon*.

[121] Sakeena M., Fa M., Za G., Mm K. (2010). Formulation and in vitro evaluation of ketoprofen in palm oil esters nanoemulsion for topical delivery. *Journal of Oleo Science*.

[122] Shakeel F. (2010). Criterion for excipients screening in the development of nanoemulsion formulation of three anti-inflammatory drugs. *Pharmaceutical Development and Technology*.

[123] Daar J., Khan A., Khan J., Khan A., Khan G. M. (2017). Studies on self-nanoemulsifying drug delivery system of flurbiprofen employing long, medium and short chain triglycerides. *Pakistan journal of pharmaceutical sciences*.

[124] Göke K., Bunjes H. (2017). Drug solubility in lipid nanocarriers: influence of lipid matrix and available interfacial area. *International Journal of Pharmaceutics*.

[125] Hamed R., Mahmoud N. N., Alnadi S. H., Alkilani A. Z., Hussein G. (2020). Diclofenac diethylamine nanosystems-loaded bigels for topical delivery: development, rheological characterization, and release studies. *Drug Development and Industrial Pharmacy*.

[126] Alam M. S., Algahtani M. S., Ahmad J. (2020). Formulation design and evaluation of aceclofenac nanogel for topical application. *Therapeutic Delivery*.

[127] Sindi A. M., Hosny K. M., Alharbi W. S. (2021). Lyophilized composite loaded with meloxicam-peppermint oil nanoemulsion for periodontal pain. *Polymers*.

[128] Assali M., Zohud N. (2021). Design of multicomponent indomethacin‐paracetamol and famotidine loaded nanoparticles for sustained and effective anti‐inflammatory therapy. *Drug Development Research*.

[129] Motawea A., Borg T., Tarshoby M., Abd El-Gawad H. (2017). Nanoemulsifying drug delivery system to improve the bioavailability of piroxicam. *Pharmaceutical Development and Technology*.

[130] Abd E., Benson H., Mohammed Y., Roberts M., Grice J. (2019). Permeation mechanism of caffeine and naproxen through in vitro human epidermis: effect of vehicles and penetration enhancers. *Skin Pharmacology and Physiology*.

[131] Smail S. S., Ghareeb M. M., Omer H. K., Al-Kinani O. A. A., Alany R. G. (2021). Studies on surfactants, cosurfactants, and oils for prospective use in formulation of ketorolac tromethamine ophthalmic nanoemulsions. *Pharmaceutics*.

[132] Kumar R., Singh A., Garg N., Siril P. F. (2018). Solid lipid nanoparticles for the controlled delivery of poorly water soluble non-steroidal anti-inflammatory drugs. *Ultrasonics Sonochemistry*.

[133] Chen B.-H., Stephen Inbaraj B. (2019). Nanoemulsion and nanoliposome based strategies for improving anthocyanin stability and bioavailability. *Nutrients*.

[134] Solans C., Izquierdo P., Nolla J., Azemar N., Garcia-Celma M. J. (2005). Nano-emulsions. *Current Opinion in Colloid and Interface Science*.

[135] Primo F. L., Michieleto L., Rodrigues M. A. (2007). Magnetic nanoemulsions as drug delivery system for Foscan®: skin permeation and retention in vitro assays for topical application in photodynamic therapy (PDT) of skin cancer. *Journal of Magnetism and Magnetic Materials*.

[136] Müller-Goymann C. C. (2004). Physicochemical characterization of colloidal drug delivery systems such as reverse micelles, vesicles, liquid crystals and nanoparticles for topical administration. *European Journal of Pharmaceutics and Biopharmaceutics*.

[137] Khanna V., Singh S., Singh Baghel D., Kaur B., Nayar A., Ghimire R. (2018). Nanoemulsions–present and future perspective-an overview. *Journal of Emerging Technologies and Innovative Research*.

[138] Thiagarajan P. (2011). Nanoemulsions for drug delivery through different routes. *Research in Biotechnology*.

[139] Shakeel F., Ramadan W. (2010). Transdermal delivery of anticancer drug caffeine from water-in-oil nanoemulsions. *Colloids and Surfaces B: Biointerfaces*.

[140] Mansour H. M., Rhee Y. S., Wu X. (2009). Nanomedicine in pulmonary delivery. *International Journal of Nanomedicine*.

[141] Liu F., Yang J., Huang L., Liu D. (1996). Effect of non-ionic surfactants on the formation of DNA/emulsion complexes and emulsion-mediated gene transfer. *Pharmaceutical Research*.

[142] Yi S. W., Yune T. Y., Kim T. W. (2000). A cationic lipid emulsion/DNA complex as a physically stable and serum-resistant gene delivery system. *Pharmaceutical Research*.

[143] Liu C.-H., Yu S.-Y. (2010). Cationic nanoemulsions as non-viral vectors for plasmid DNA delivery. *Colloids and Surfaces B: Biointerfaces*.

[144] Bivas-Benita M., Oudshoorn M., Romeijn S. (2004). Cationic submicron emulsions for pulmonary DNA immunization. *Journal of Controlled Release*.

[145] Ganta S., Deshpande D., Korde A., Amiji M. (2010). A review of multifunctional nanoemulsion systems to overcome oral and CNS drug delivery barriers. *Molecular Membrane Biology*.

[146] Dhahir R. K., Al-Nima A. M., Al-Bazzaz F. (2021). Nanoemulsions as ophthalmic drug delivery systems. *Turkish Journal of Pharmaceutical Sciences*.

[147] Pardridge W. M. (1999). Non-invasive drug delivery to the human brain using endogenous blood–brain barrier transport systems. *Pharmaceutical Science and Technology Today*.

[148] Kumar M., Misra A., Babbar A. K., Mishra A. K., Mishra P., Pathak K. (2008). Intranasal nanoemulsion based brain targeting drug delivery system of risperidone. *International Journal of Pharmaceutics*.

[149] Mistry A., Stolnik S., Illum L. (2009). Nanoparticles for direct nose-to-brain delivery of drugs. *International Journal of Pharmaceutics*.

[150] Csaba N., Garcia-Fuentes M., Alonso M. J. (2009). Nanoparticles for nasal vaccination. *Advanced Drug Delivery Reviews*.

[151] Rinaldi F., Maurizi L., Forte J. (2021). Resveratrol-loaded nanoemulsions: in vitro activity on human T24 bladder cancer cells. *Nanomaterials*.

